# Leukocyte Population Dynamics and Detection of IL-9 as a Major Cytokine at the Mouse Fetal-Maternal Interface

**DOI:** 10.1371/journal.pone.0107267

**Published:** 2014-09-26

**Authors:** Mohamed Habbeddine, Philippe Verbeke, Sonia Karaz, Pierre Bobé, Colette Kanellopoulos-Langevin

**Affiliations:** 1 Laboratory of Inflammation, Gestation and Autoimmunity, Jacques Monod Institute, CNRS and University Paris-Diderot, Paris, France; 2 Genetics and development of the cerebral cortex, Jacques Monod Institute, CNRS and University Paris-Diderot, Paris, France; Queen’s University, Canada

## Abstract

Despite much interest in the mechanisms regulating fetal-maternal interactions, information on leukocyte populations and major cytokines present in uterus and placenta remains fragmentary. This report presents a detailed and quantitative study of leukocyte populations at the mouse fetal-maternal interface, including a comparison between pregnancies from syngeneic and allogeneic crosses. Our results provide evidence for drastic differences not only in the composition of leukocyte populations in the uterus during pregnancy, but also between uterine and placental tissues. Interestingly, we have observed a significant decrease in the number of myeloid Gr1+ cells including monocytes, and myeloid CD11c+ cells including DCs in placenta from an allogeneic pregnancy. In addition, we have compared the expression levels of a panel of cytokines in non-pregnant (NP) or pregnant mouse uterus, in placenta, or in their isolated resident leukocytes. Qualitative and quantitative differences have emerged between NP, pregnant uterus and placenta. Unexpectedly, IL-9 was the major cytokine in NP uterus, and was maintained at high levels during pregnancy both in uterus and placenta. Moreover, we have found that pregnancy is associated with an increase in uterine IL-1a and a significant decrease in uterine G-CSF and GM-CSF. Comparing allogeneic versus syngeneic pregnancy, less allogeneic placental pro-inflammatory cytokines CCL2 (MCP-1), CXCL10 (IP-10) and more IL1-α in whole uterus was reproducibly observed. To our knowledge, this is the first report showing a detailed overview of the leukocyte and cytokine repertoire in the uterus of virgin females and at the fetal-maternal interface, including a comparison between syngeneic and allogeneic pregnancy. This is also the first evidence for the presence of IL-9 in NP uterus and at the maternal-fetal interface, suggesting a major role in the regulation of local inflammatory or immune responses potentially detrimental to the conceptus.

## Introduction

Understanding the intricate mechanisms regulating mammalian fetal-maternal interactions has remained a challenging goal for biologists for several decades [1]. During pregnancy, the maternal immune system faces the double task of protecting itself and the conceptus against pathogens, as well as preventing the rejection of the semi-allogeneic feto-placental unit [2].

Although the murine and human placentae differ in their detailed structure [3], we have chosen the mouse model because the human and mouse immune systems have numerous similarities, and the two species share a hemochorial placentation as well as many functional mechanisms and regulations at the fetal-maternal interface. For ethical and practical reasons, human samples are obtained only at early stages of pregnancy or after delivery, or they come from spontaneous or therapeutic abortions, possibly introducing a bias in the study of various cell populations.

Despite much interest in the mechanisms regulating fetal-maternal interactions in normal or pathological situations in mice, information on the different leukocyte populations at the fetal-maternal interface remains fragmentary. During pregnancy, the uterus undergoes drastic modifications in order to allow the implantation of the embryo, a necessary step for its survival and development. Concomittantly, the uterine endometrium is transformed into decidua around the implantation site. It is induced under the influence of many factors including LIF and progesterone [4]. Decidualisation consists of tissue remodeling and angiogenesis associated with massive leukocyte infiltration [5], [6], primarily uterine NK cells [7]–[11].

Stromal and immune cells present at the fetal-maternal interface are suspected to play many important roles in the maintenance and regulation of pregnancy [2], [12]–[15].

Numerous cytokines have been shown also to play important roles in decidualisation and placentation, as well as maintenance of pregnancy [6]. Disturbance of the normal expression or balance of these cytokines could result in complete or partial failure of implantation and abnormal placenta formation in mice or humans, leading to pregnancy failure [4]. During later phases of gestation, a Th2 cytokine profile, responsible for the down-modulation of pro-inflammatory and cytotoxic responses, has been assumed to be in favor of the success of pregnancy [16]–[18]. This cytokine bias at the fetal-maternal interface is thought to prevent fetal harm from inflammatory responses. Likewise, the immunosuppressive environment would be preventing maternal adaptive cytotoxic responses against the fetal-placental unit. Among Th2 cytokines, IL-10 is immunosuppressive on Th1 cells and acts primarily on antigen presenting cells (APCs) to induce T cell anergy or IL-10 producing Tregs, most of which also produce TGFβ. TGFβ is a pleiotropic cytokine with potent immunosuppressive activity on the majority of the cellular components of immune responses. Both IL-10 and TGFβ have been shown to be present in important amounts at the fetal-maternal interface in mice and humans [6], [19]–[24], where they are assumed to play an immunoregulatory role. Recently, an important chemokine gene silencing mechanism has been described in decidual stromal cells, limiting T cell access to the maternal-fetal interface [15]. An alternative mechanism deciphered by the same group showed that dendritic cells in the decidua are trapped *in situ* and prevented from migrating to the uterine lymphatic vessels to reach the draining lymph nodes [25].

The present report provides novel detailed information on the different leukocyte populations present at the fetal-maternal interface from post-implantation to post-partum, including a comparison between syngeneic and allogeneic pregnancies. Our results bring evidence for drastic changes, in uterine leukocyte populations during pregnancy compared to a NP uterus, as well as major differences in leukocyte distributions between uterus and placenta. In addition, more subtle differences can be detected when comparing syngeneic to allogeneic uterus and placenta, such as a significantly decreased number of myeloid GR1+ cells including monocytes and myeloid CD11c+ cells including DCs. Moreover, we have analyzed a panel of pro and anti-inflammatory cytokines and chemokines produced in the NP or syngeneic or allogeneic pregnant uterus, and placenta. We have performed the same analyses at the level of uterine and placental resident leukocytes. Interesting qualitative and quantitative differences have emerged between non-pregnant (NP) and pregnant uterus, and placenta, as well as between syngeneic or allogeneic pregnancies. As expected, IL-10 was present in large amounts, but surprisingly, out of the panel of cytokines tested, IL-9 was present in largest quantities, in NP and pregnant uterus and placenta. To our knowledge, this is the first report demonstrating the presence of important amounts of IL-9 at the fetal-maternal interface. In addition, we observed much lower levels of uterine G-CSF and GM-CSF during pregnancy. When we compared allogeneic and syngeneic crosses, the most striking feature was a lower level of pro-inflammatory cytokines CCL2 and CXCL10 in allogeneic pregnancy. These novel findings are discussed in the light of recent findings on the role of IL-9 in the induction of immunological tolerance to allografts.

## Results

### Leukocyte preparation from non-pregnant (NP) or pregnant mouse uterus and placenta

Few studies have described in detail the different leukocyte populations at the fetal-maternal interface, in part due to the difficulty to prepare significant numbers of viable immune cells from the placenta and uterus. In order to better understand the different roles of the maternal immune system at the fetal-maternal interface, we first established an optimal protocol to extract leukocytes from uterus of NP or pregnant mice and from placenta. We followed the evolution of both uterine and placental leucocyte populations at different time points during pregnancy. We killed mice on 5.5 days *post coitum* (d*pc*) one day after implantation, on day 10.5 d*pc* during placenta formation, and on days 13.5 and 16.5 d*pc* when the placenta is fully mature and fetal-maternal immune interactions are most active. Tissues were carefully dissected and leukocytes were prepared after lengthy optimization of the protocol.

**Figure 1:**
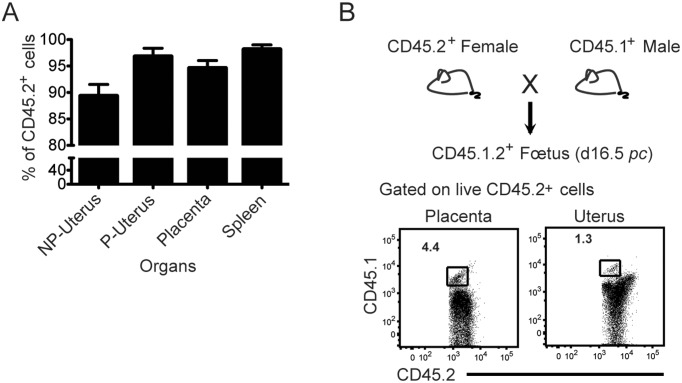
Purity of leukocytes and presence of fetal cells after enrichment from placenta and uterus (16.5 d*pc*). A. Enriched leukocyte preparations from non-pregnant (NP) uterus, pregnant (16.5 d*pc*) uterus (P) and placenta were analyzed by flow cytometry. Viable cells excluding propidium iodide were gated on the basis of forward (FSC) and side scatter (SSC) criteria. The average leukocyte purity assessed by the presence of the CD45.2 marker was 89.3% for NP uterus, 97% for pregnant uterus, and 95% for placenta, compared to 98% for the spleen (positive control for the assessment of purity) from the same mice. Analyses were performed on at least 3 animals per group B. CD45.2+ B6 females were crossed with CD45.1+ B6 males. CD45+ leukocyte populations from the uterus and placenta were analyzed by flow cytometry on day 16.5 *pc*. The detection of CD45.1+2+ cells revealed the presence of a small percentage (<5%) of fetal leukocytes. The experiment has been repeated 3 times.

Leukocytes from uterus and placenta (16.5 d*pc)* were enriched on a Percoll gradient (80%/40%). The leukocyte purity was assessed using the CD45.2 marker, which stained 89 to 97% of cell preparations from the different tissues, with classical spleen cell preparations included as positive controls (Figure 1A). Pregnant uterine leukocyte preparations reached the same purity level as spleen cells from the same mouse.

In order to assess the contribution of fetal lymphoid cells versus maternal cells in these populations, we crossed a CD45.2+ B6 female with a CD45.1+ B6 male, and we checked for the presence of leucocytes of fetal origin bearing both CD45.2+ and CD45.1+ cell markers. Figure 1B shows that, on day 16.5 of gestation, a very small percentage of fetal cells was detectable in viable cell preparations from uterus (1.3%) and placenta (4.3%).

### Leukocyte population scatter profiles change drastically during pregnancy

**Figure 2:**
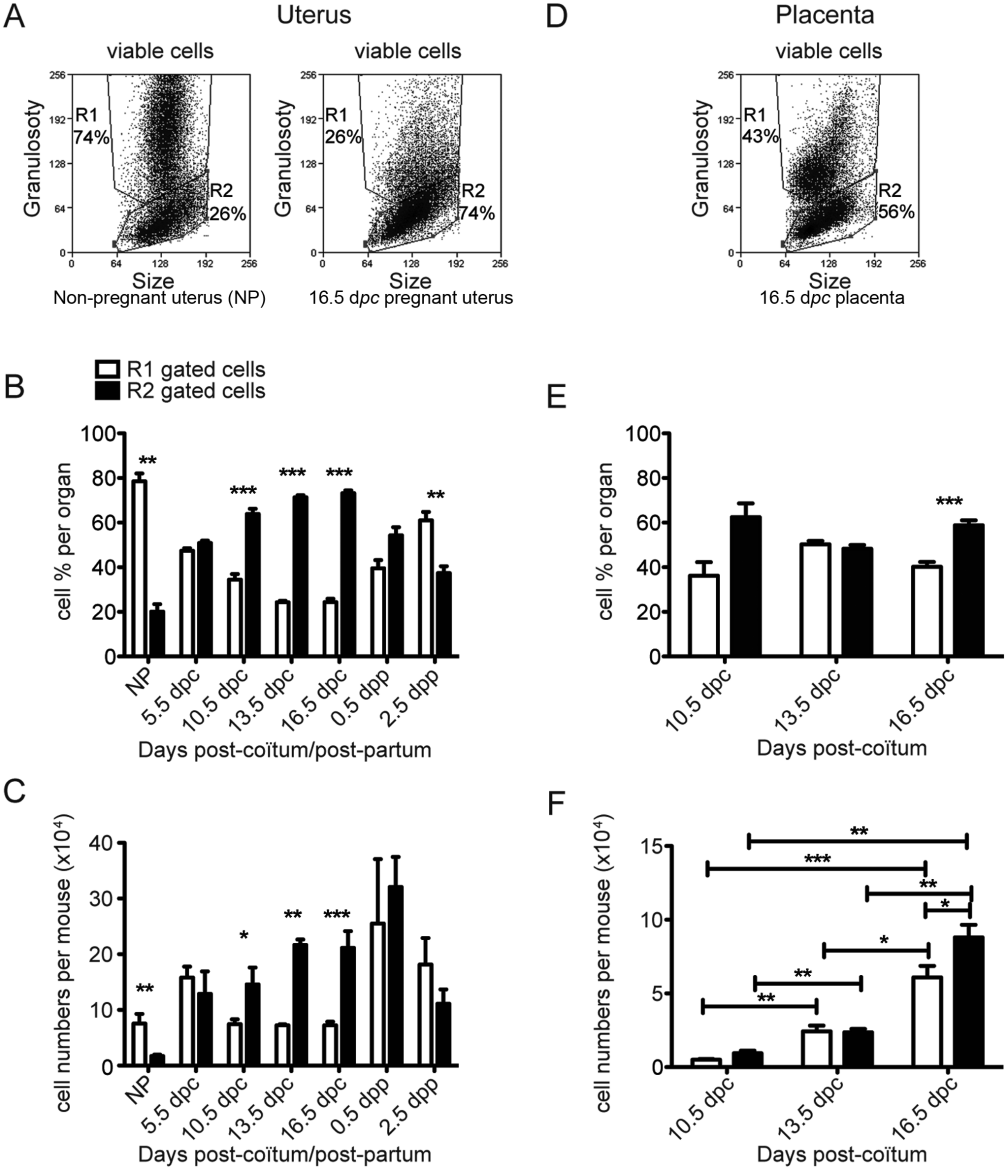
Drastic changes in the scatter distribution of leukocytes from NP, or pregnant uterus, and placenta at various stages of a syngeneic pregnancy. The same protocol as Figure 1 was followed. Viable cells excluding propidium iodide were gated on the basis of forward (FSC) and side scatter (SSC) criteria, from uterus (A) or placenta (D). Mean percentages (B, E) and total cell numbers (C, F) in « granular » (R1 gate, white bars) or « lymphoïd/monocytoïd » (R2 gate, black bars) are presented. NP uteri were pooled from 4 to 15 mice in all phases of œstrus cycle and the experiment was repeated 6 times. At each stage of pregnancy or post-partum, the data were collected from 4 to 14 mice assayed in 2 to 9 separate experiments. (*) p≤0.05, (**) p≤0.005, (***) p≤0.001.

On the basis of size and granulosity measured by flow cytometry (forward and side scatter profiles, respectively), two viable single cell populations could be distinguished. In a typical experiment presented in Figure 2A (left dot plot), larger and more granular cells gated in R1 represented 74% of the cell preparation from a pool of NP uterus and presented a very heterogeneous population, both in size and granulosity, even after doublet elimination. The minor cell population defined by the R2 gate, much less granular than cells gated in R1, contained lymphoid and monocytic cells, as expected. In this particular experiment, it represented 26% of the total. We have analysed the evolution of these two uterine leukocyte populations based on scatter profiles, during a syngeneic B6xB6 pregnancy, on 5.5, 10.5, 13.5 and 16.5 d*pc*, and 0.5, 2.5 and 5.5 days *post partum* (d*pp*). The whole uterus was gently separated from embryos, extra-embryonic tissues and placentas to be used for the preparation of leukocytes, except on day 5.5 *pc*. when the whole uterus was taken along with recently implanted embryos. As pregnancy progressed, we observed a clear and significant diminution of highly granular uterine leukocytes present in the R1 gate (Figure 2A, right dot plot, d16.5 *pc*), both in frequency (Figure 2A and B) and cell numbers (Figure 2C), with a minimum between 13.5–16.5 d*pc* in frequency (25%) and between 10.5–16.5 d*pc* in number. We observed, in parallel, significantly more uterine leukocytes present in the R2 gate between 13.5–16.5 d*pc* (72%). Interestingly, the distribution pattern appears to return to the NP R1/R2 ratio within a few days after delivery (Figure 2B and 2C).

In the same experiment, placental leukocytes were analysed on the same scatter basis starting from day 10.5 *pc,* when placenta can be easily distinguished and dissected from surrounding tissues. Again, we could clearly observe two cell populations. The more granular population gated in R1 appeared more homogeneous than in uterus and represented 43% of placental leukocytes (Figure 2D). The cell population in the R2 gate contained 56% of the leukocyte preparation (Figure 2D). During the course of pregnancy, the percentages of R1 versus R2 placental leukocytes remained around 50% with the exception of 16.5 d*pc* when R2-gated cells were significantly more frequent (Figure 2E, p≤0.001) and more numerous (Figure 2F, p≤0.05). In addition, both R1 and R2 placental cell numbers were steadily increased from day 10.5 until 16.5 d*pc,* in parallel to placental maturation and increase in volume (p<0.005 for R2 and p<0.0005 for R1 gates, Figure 2F).

Thus, in uterus and placenta, pregnancy is associated with drastic changes in various leukocyte size and granulosity, with cell frequency and numbers significantly altered during the course of pregnancy.

### Immune cell populations present in the uterus of NP or pregnant mice, and in placenta

In order to decipher the leukocyte populations present in the pregnant or NP uterus, we stained the cell preparations with a panel of lineage specific fluorochrome-coupled monoclonal antibodies (mAbs) anti-Gr-1, CD11b, CD11c, TCRβ chain, NK1.1, CD4, CD8, CD19, B220 (Table 1) and analyzed them by flow cytometry. We chose to perform experiments on 16.5 d*pc* when the placenta is fully mature, and the effects of pregnancy on uterine leukocyte repartition are more pronounced, as indicated above in the results presented in Figure 2.

**Table 1:** Antibody and dilutions used for flow cytometry staining.

Specificity	Clone	Reference	Coupling	Dilution used 1/n	Isotype	Supplier
**CCR2**	475301	FAB5538A	APC	100	Rat IgG2b	R&D Systems
**CD3ε**	145-2C11	1535-02	FITC	200	Armenian Hamster IgG	Southern Biotechnology
**CD3ε**	17A2	56-0032-82	A700	400	Rat IgG2b, κ	eBioscience
**CD4**	GK1.5	1540-09	PE	400	Rat IgG2b, κ	Southern Biotechnology
**CD4**	RM4-5	12-0042	PE	400	Rat IgG2a, κ	Southern Biotechnology
**CD4**	RM4-5	550954	PerCP-Cy5.5	1000	Rat IgG2a, κ	BD bioscience
**CD8α**	53-6.7	1550-02 S	FITC	400	Rat IgG2a, κ	eBioscience
**CD8α**	53-6.7	11-0081	FITC	400	Rat IgG2a, κ	eBioscience
**CD8α**	53-6.7	25-0081-82	PE-Cy7	800	Rat IgG2a, κ	eBioscience
**CD11b**	M1/70	48-0112-82	eFluor450	400	Rat IgG2b, κ	eBioscience
**CD11b**	M1/70	557672	Alexa488	400	Rat IgG2b, κ	BD Pharmingen
**CD11b**	M1/70	11-0112-85	FITC	400	Rat IgG2b, κ	eBioscience
**CD11b**	M1/70	12-0112	PE	400	Rat IgG2b, κ	eBioscience
**CD11c**	N418	25-0114-82	PE-Cyanine7	400	Armenian Hamster IgG1, κ	eBioscience
**m CD11c**	HL3	558079	PE-Cy7	500	Armenian Hamster IgG1, λ2	BD bioscience
**CD16/CD32**	2.4G2	553-142	Purified	200	Rat IgG2b, κ	BD bioscience
**CD19**	1D3	553785	FITC	400	Rat IgG2a, κ	BD bioscience
**m CD24**	M1/69	17-0242-82	APC	1000	Rat IgG2b, κ	eBioscience
**m CD45.1**	A20	560520	V450	500	Mouse IgG2a, κ	BD bioscience
**m CD45.2**	104	558702	APC	500	Mouse IgG2a, κ	BD bioscience
**m CD45.2**	104	553771	biotin	800	Mouse IgG2a, κ	BD bioscience
**m CD45.2**	104	560697	V450	300	Mouse IgG2a, κ	BD bioscience
**m CD45.2**	104	11-0454-81	FITC	200	Mouse IgG2a, κ	eBioscience
**F4/80**	BM8	47-4801-82	APC-eFluor780	200	Rat IgG2a, κ	eBioscience
**Gr-1**	RB6-8C5	553125	Biotin	400	Rat IgG2b, κ	BD bioscience
**Gr-1**	RB6-8C5	553128	PE	400	Rat IgG2b, κ	BD bioscience
**Ly-6C**	AL-21	553104	FITC	1000	Rat IgM, κ	BD bioscience
**Ly6G**	1A8	551461	PE	600	Rat IgG2a, κ	BD bioscience
**MHC II (I-A/I-E)**	M5/114.15.2	56-5321-82	A700	400	Rat IgG2b, κ	eBioscience
**MHC II (I-A/I-E)**	2G9	558593	PE	400	Rat IgG2a, κ	BD bioscience
**MHC II (I-A/I-E)**	AF6-120.1	553552	PE	400	Mouse IgG2a, κ	BD bioscience
**NK1**-**1**	PK136	557391	PE	400	Mouse IgG2a, κ	BD bioscience
**NK1**-**1**	PK136	12-5941-82	PE	400	Mouse IgG2a, κ	eBioscience
**TCRβ**	H57-597	13-5961-85	biotin	500	Armenian Hamster IgG	eBioscience
**Streptavidin-PE-Cy7**	NA	557598	PE-Cyanine7	500	NA	BD bioscience
**Streptavidin-APC**	NA	17-4317-82	APC	400	NA	eBioscience
**Syrian Hamster IgG**	NA	007-000-003	Purified	200	Whole IgG molecules	Jackson ImmunoResearch
**SYTOX Blue dead cell stain**	NA	S34857	NA	5000	NA	Life technologies

NA: not applicable.

**Figure 3:**
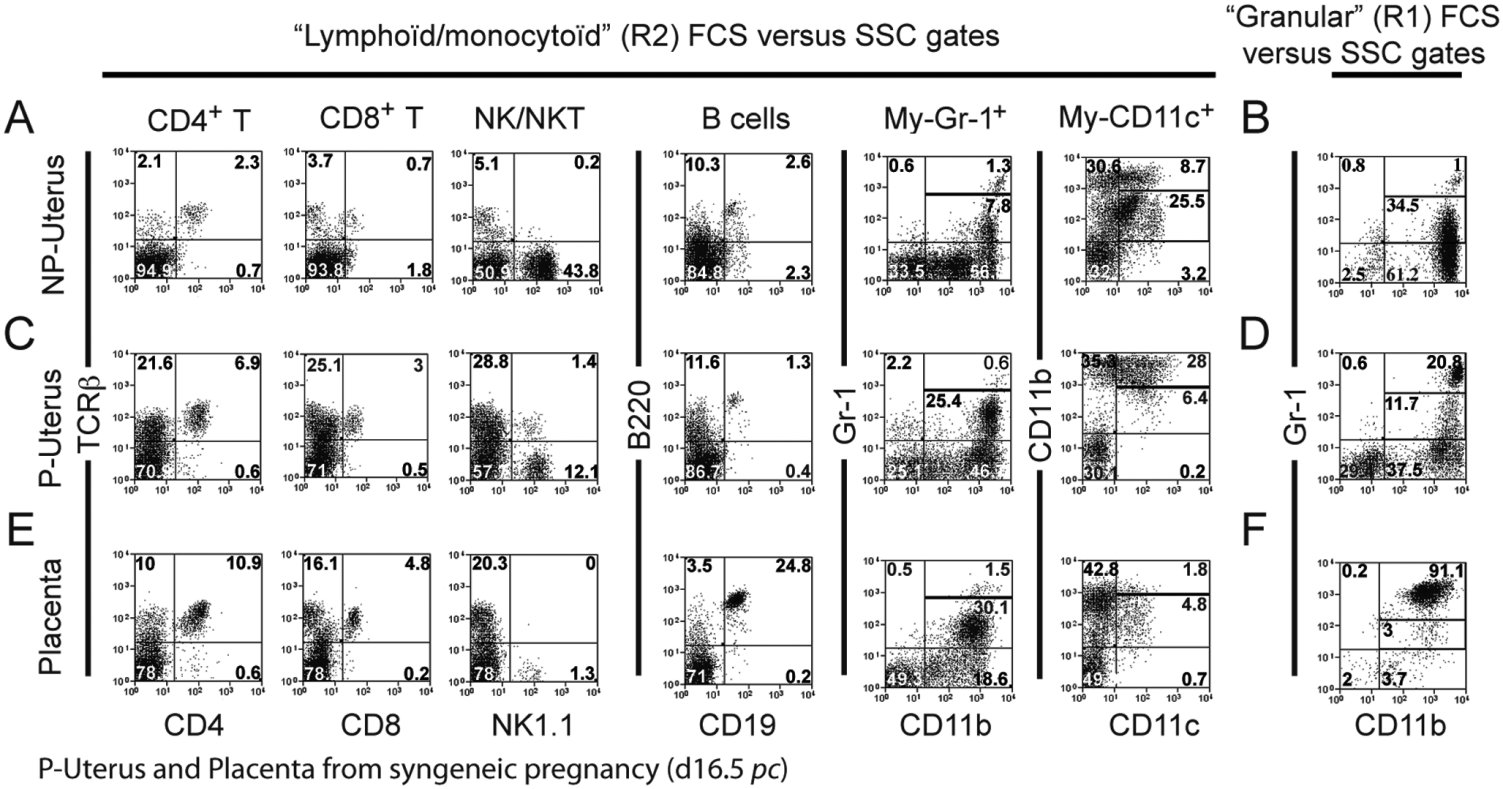
Analyses of immune cell populations present in the uterus of NP or pregnant mice, and in placenta (16.5 d*pc*). Only viable cells excluding propidium iodide were analysed. Enriched leukocytes from NP (A,B) or pregnant (16.5 d*pc*) uterus (C,D) and placenta (E,F) were analysed by flow cytometry. R2-gated cells (left, cf. Figure 2) were analysed on the basis of the following cell surface markers: TCRβ^+^/CD4^+^ (CD4 T cells); TCRβ^+^/CD8^+^ (CD8 T cells); NK1.1^+^/TCRβ^−^ (NK cells); TCRβ^+^/NK1.1^+^ (NKT cells); CD19^+^/B220^+^ (B cells); Gr1^+^/CD11b^+^ (myeloid Gr1+ cells including monocytes); Gr1^−/^CD11c^+^/CD11b^Hi/low^ (myeloid CD11c+ cells including DCs). R1-gated cells (right, cf. Figure 2) were analysed on the basis of the following cell surface markers: Gr1^+^/CD11b^+^ (Granulocytes); CD11b^Hi^/Gr1^+^/Gr1^+/−/^CD11c^+/−^ (Highly granulosity cells or HGC). The results are representative from a typical experiment of a pool of 7 mice (A, B) or from a single mouse (C, D, E, F). The experiment was repeated at least twice with 3–6 animals per assay.

A typical experiment is presented in Figure 3. As can be seen in Figures 3A and 3C, NP or pregnant uterine leukocytes within the R2 gate (cf. Figure 2A) comprised CD4 T cells (TCRβ^+^ CD4^+^), CD8 T cells (TCRβ^+^ CD8^+^), NK cells (NK1.1^+^ TCRβ^−^), NKT cells (TCRβ^+^ NK1.1^+^), B cells (CD19^+^ B220^+^), myeloid GR-1+ cells including monocytes (CD11b^+^ Gr-1^lo^), and myeloid CD11c+ cells including dendritic cells (DCs), positive for the CD11b marker with two distinct fluorescence levels (CD11b^Hi/l^°CD11c^+^ Gr-1^−^). We have completed our study with results presented in Figure S1. In these experiments, live, R2-gated CD45.2+, Gr1-, CD11b+, CD11c+ cells from uterus or placenta on day 16.5 *pc* were stained anti-F4/80, -CCR2 or -MHC class II antibodies. The cell populations present a heterogeneous level of CD11b expression from low to intermediate to high. The macrophage marker F4/80 is negative on 2/3 of cells in the placenta and half the cells in the uterus. The inflammatory monocyte marker CCR2 is expressed on 75 and 67% of cells in placenta and uterus, respectively. MHC class II molecules are present on 42% of placental cells and 65% of uterine cells with low to high expression. Thus, the R2-gated Gr-1-, CD45.2+, CD11b+, CD11c+ population is very heterogeneous and includes both DCs and monocytes/macrophages. That is why we have adopted the general terms “myeloid Gr-1+ (CD11c-) cells” and “myeloid (Gr-1-) CD11c+ cells”.

In NP uterus, both R1 and R2 gates comprised a small population of granulocytes (Gr1^Hi^ CD11b^Hi^, about 1% of gated cells) (Figures 3A and 3B). Surprisingly, the majority of R1-gated cells (60 to 80%) were much larger and more granular than “classical” granulocytes, expressing high levels of CD11b. A fraction of these cells only was Gr-1+ (Figures 2A and 3B). In addition, these highly granular cells (HGC) do not express the TCRβ chain, CD4, CD8, CD19, NK1.1 or B220 molecules (see below).

**Figure 4:**
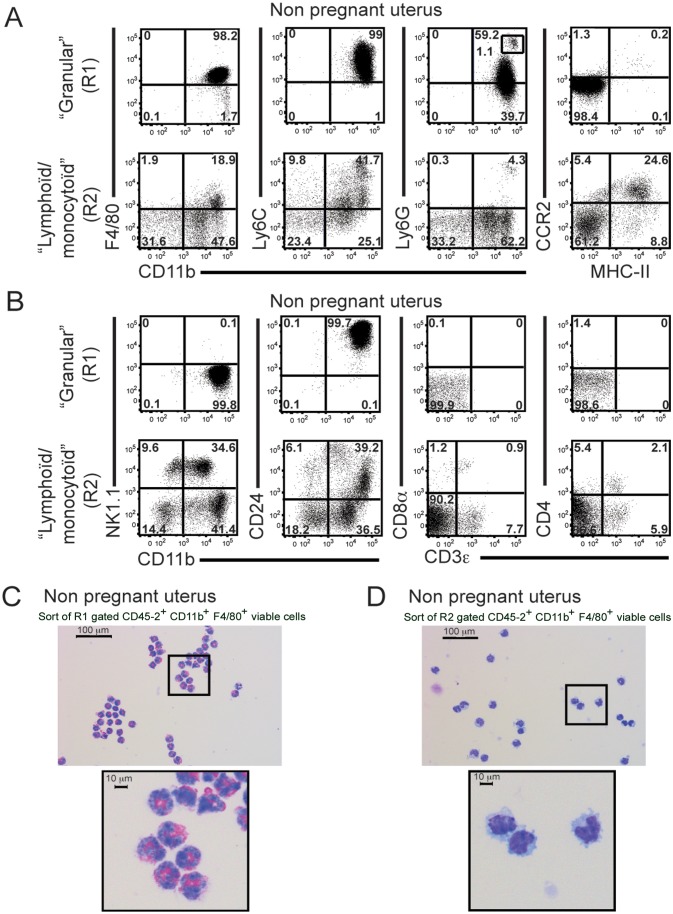
High granulosity cells in non pregnant uterus are primarily eosinophils. Viable R1- or R2- gated CD45.2+ cells from NP uterus were analysed by FACS stained for APC and granulocytes markers (CD11b, CD24, CD11b, F4/80, Ly6C, Ly6G, MHC class II and CCR2) (A, B and Table 1) and for NK and T cell markers (CD11b, NK1.1, CD3ε, CD8α and CD4) (B and Table 1). The experiment was repeated twice with 6 females each time (A, B). Viable CD45.2+, CD11b+, F4/80+ R1 (C) or R2 (D) cells were sorted by flow cytometry and spun onto Super + glass microscope slides using a Cytospin cytofuge. Cells were fixed with methanol and stained with Wright-Giemsa. Pictures were taken on a Nikon H600L microscope equipped with a DS-Fi2-Nikon camera (C, D). The experiment was performed twice with at least 5 females each time.

We analyzed these cells further, and our results are presented in Figure 4. As shown in Figure 4A, R1 gated cells from NP uterus were nearly all CD11b high, F4/80+ and Ly6C+ (98.3 and 99%, respectively), 60.4% were Ly6G+ (Gr1+), including 1% CD11bhi, Ly6Ghi cells, probably polymorphonuclear neutrophils. Almost all cells (98.2%) were CCR2- and MHC class II-negative, two markers known to be expressed on monocytes and APC, respectively [26], [27]. These data show that HGC are not APC (including B cells, DCs and monocytes). Figure 4B confirmed our previous results that R1 gated cells were NK1.1-, CD3ε-, CD4- and CD8-negative, but 99.7% CD24+ (a marker of mature granulocytes in this gate). Moreover, fewer than 5% of R1-gated cells were stained weakly by DBA (Figure S2). These results exclude αβT cells, mast cells and uterine NK cells.

In contrast, and as expected, cells from the R2 lymphoid/monocytoid gate expressed heterogeneous levels of CD11b and Ly6C, less than 5% were Ly6G+cells (possibly a small neutrophil contamination from gate R2), 24.6% CCR2+, MHC class II+ (migratory monocytes/DCs) and 8.8% MHC class II + cells (Figure 4A). As shown in Figure 4B, 18.9% cells were positive for both macrophage markers CD11b and F4/80. R2-gated cells also contained CD11b+, NK1.1+ cells (34.6%), 9.6% CD11b-, NK1.1+ cells, 39.2% CD11b+,CD24+ cells (granulocytes and B cells), and small percentages of CD8+ and CD4+ T cells (0.9 and 2.1%, respectively).

When CD45.2+, CD11b+, F4/80+ cells from the granular R1 gate were sorted on slides and stained with Wright/Giemsa, a great majority of cells were large granular polymorphonuclear leukocytes with eosinophilic granules *i.e.* eosinophils (Figure 4C). The same sort from R2 cells yielded a majority of monocytes/macrophages (Figure 4D).

**Figure 5:**
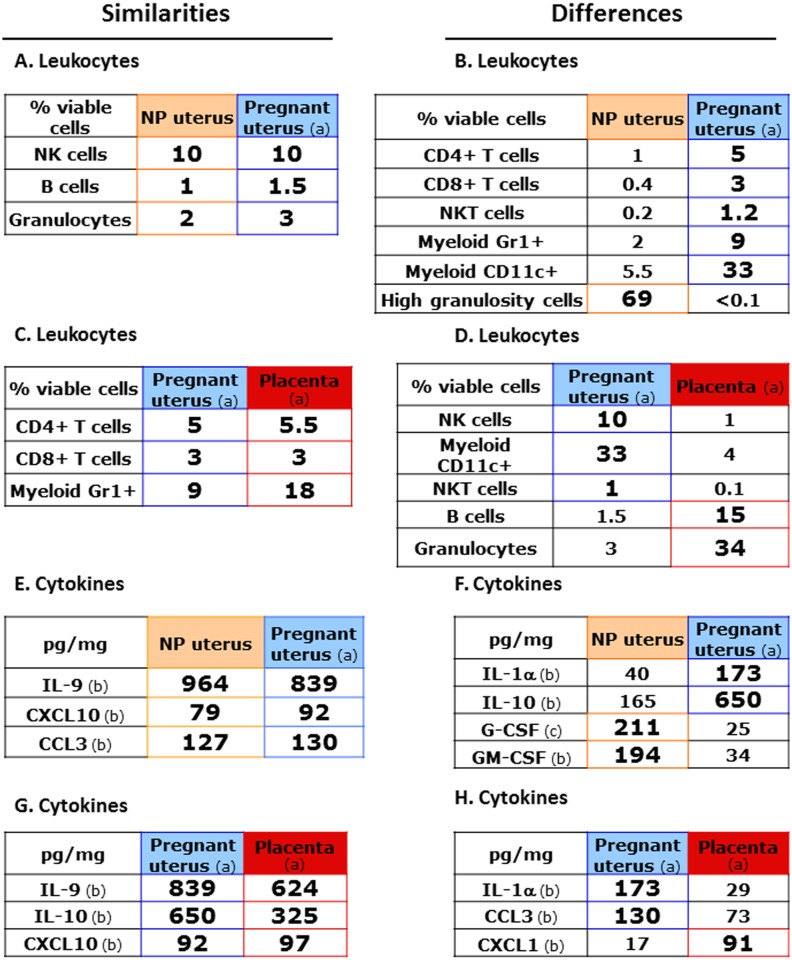
Similarities and differences in leukocyte subpopulation frequencies and cytokine/chemokine production between NP, pregnant uterus and placenta. **A, B, C, D**: comparison of **leukocyte subpopulation frequencies** **E,** **F,** **G,** **H**: comparison of **cytokine/chemokine productions** (a) syngeneic pregnancy (day 16.5 *pc*) (b) cytokines/chemokines from leukocyte-enriched preparations (c) cytokines/chemokines from whole uterus preparations Similarities referred to comparable cell percentages or cytokine/chemokine quantities, varying at most by a factor of 2. Differences corresponded to large statistically significant differences by factors ranging from 4 to 700.

In order to synthesise the results on cell populations, data was presented in Figure 5. “Similarities and differences” in leukocyte frequencies between NP and pregnant uterus have been highlighted in Figure 5A and 5B. Similarities referred to comparable cell percentages, varying at most by a factor of 2. Differences corresponded to large statistically significant differences by factors ranging from 4 to 700.

Interestingly, R2-gated B cell, NK cell and granulocyte frequencies were not affected by pregnancy in uterus (Figure 5A, Figure 3A and C). In contrast, among R2-gated cells, the percentages of CD4 and CD8 T cell, NKT cells, myeloid GR1+ cells and CD11b^lo/hi^ myeloid CD11c+ cells are all increased by a factor of 2 to 7, while NK cells were decreased by a factor of 3.6. High granulosity cells (HGC), the most abundant in NP uterus (69% of R1-gated cells), became barely detectable during pregnancy (<0.1% of R1+R2 leukocytes) (Figure 5B).

Neutrophils (SSC-A^Hi^ CD11b^Hi^ Gr-1^Hi^) were much more numerous in pregnant uterus (20.8% of R1 cells) than in NP uterus (1% of R1 cells) (Figure 3B and D).

In the placenta, as shown in Figure 3E, CD4 T cells were 10.9% of R2-gated cells, CD8 T cells 4.8%, and NK cells represented the smallest population with 1.3% of cells in the R2 gate. NKT cells were below detection level in this particular experiment. Surprisingly, R2-gated cells comprised mainly 24.8% B cells and 30.1% myeloid Gr1+ cells. Myeloid CD11c+ cells (including DCs) represented 6.6%. The R1 gate mainly contained granulocytes, 91.1% (Figure 3F).

### Quantitative analysis of leukocyte population variations in a pool of B6 NP versus pregnant uterus, including a comparison between syngeneic and allogeneic pregnancy

**Figure 6:**
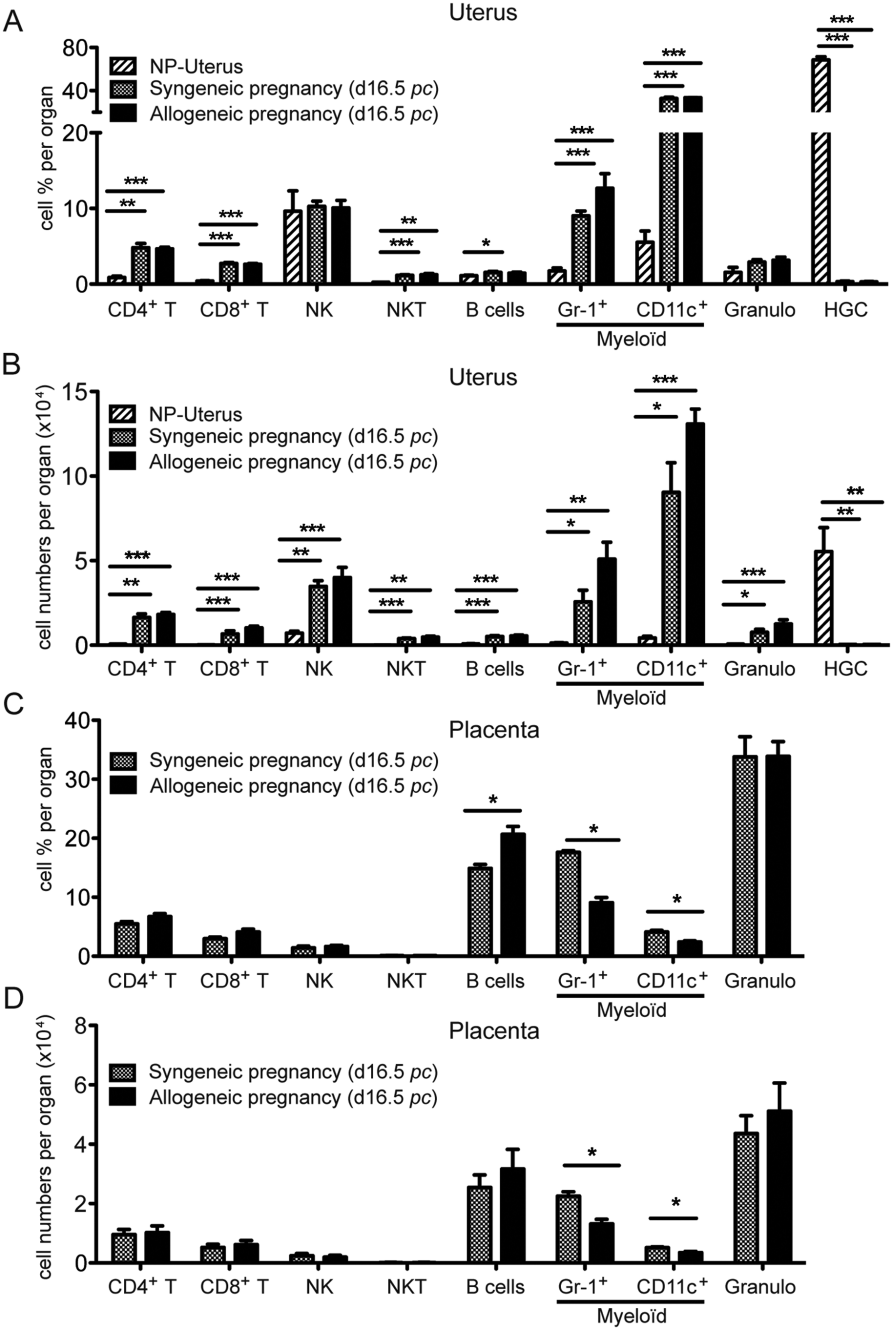
Comparison of syngeneic versus allogeneic pregnancies from the mean percentages and cell numbers of uterine and placental leukocyte populations (day 16.5 *pc*). Mean cell percentages among all viable leukocytes (A and C) and corresponding mean cell numbers, per uterus (B) or per placenta (D), were measured by flow cytometry in leukocyte subpopulations from NP-uterus (striped bars, A–B), compared to the uterus from syngeneic (grey bars, A–B) or allogeneic pregnancies (black bars, A–B), and in placenta from syngeneic (grey bars) or allogeneic pregnancies (black bars, C–D), on day 16.5 *pc*. NP-uteri were pooled from 4 to 15 mice in all phases of estrus cycle, in at least 3 different experiments. For each experiment, 3 to 9 placentas or implantation sites were collected. The experiment was performed 4 times. (*) p≤0.05, (**) p≤0.005, (***) p≤0.001.

As shown in Figures 6A and 6B, most uterine leukocyte populations were significantly increased in percentages (of all R1+R2 gated leukocytes) and numbers during pregnancy, compared to the NP state. In a pregnant uterus, myeloid CD11c+ cells were the most frequent and numerous leukocyte populations (around 34%) followed by NK (10%), myeloid GR1+ cells (around 10%), αβT cells (7%), B (1 to 2%) and NKT cells (1%). Remarkably, HGC have virtually disappeared from the pregnant uterus, as seen previously in Figure 2A.

Another striking feature is that no significant differences are found in uterine populations (frequencies or numbers), when allogeneic and syngeneic pregnancies are compared (Figures 6A and 6B).

### Comparative analysis of immune cell populations present in placenta during syngeneic and allogeneic pregnancy

In placenta, we used the same gating strategy as in uterus. All cell populations were present at 16.5 d*pc* in different proportions except for HGC, which were nearly absent (Figures 6C and 6D). In placentas from the same syngeneic pregnant mice, the cell distributions were significantly different from the uterus. The most frequent and numerous cell types among all leukocytes were granulocytes (33%), followed by B cells (15 to 20%), myeloid GR1+ cells (17 to 9%), αβT cells (8 to 11%) and myeloid CD11c+ cells (4 to 2%). NK were a small minority of cells (1 to 2%), and NKT cells were barely detectable, again in contrast to the uterus (Figure 6). Placental leukocyte populations (assessed in individual animals) were also compared in syngeneic versus allogeneic pregnancy (Figure 6C and 6D). Our results showed a significant decrease in frequency and numbers of myeloid GR1+ cells (from 17 to 9%, p<0,05), and myeloid CD11c+ cells (from 4 to 2% p<0,05) in allogeneic pregnancy. In contrast, B cell percentages were significantly increased in the allogeneic situation (from 15 to 20% p<0,05) but differences in number were not significant.

In conclusion, during the second half of gestation, nearly all leukocyte populations are present in the uterus and placenta, but they are distributed in significantly different numbers and frequencies in both organs. As shown in Figure 5C, similarities between leukocyte populations include comparable frequencies of CD4 and CD8 T cells, while percentages of myeloid Gr1+ cells comprising monocytes/macrophages are also in the same range. In contrast, NK, NKT cells and myeloid CD11c+ cells are much more frequent in pregnant uterus than in placenta, while B cells and granulocytes are much more abundant in placenta than in uterus (Figure 5D).

Comparing allogeneic to syngeneic pregnancies, contrary to uterus, differences can be detected in placental leukocyte proportions and numbers. It thus seems that the recognition of paternal alloantigens has more detectable consequences on placental than uterine leukocytes, with a significant decrease in placental myeloid GR1+ cells and myeloid CD11c+ cells.

### Quantification of uterine and placental cytokines and chemokines in syngeneic pregnancy

**Figure 7:**
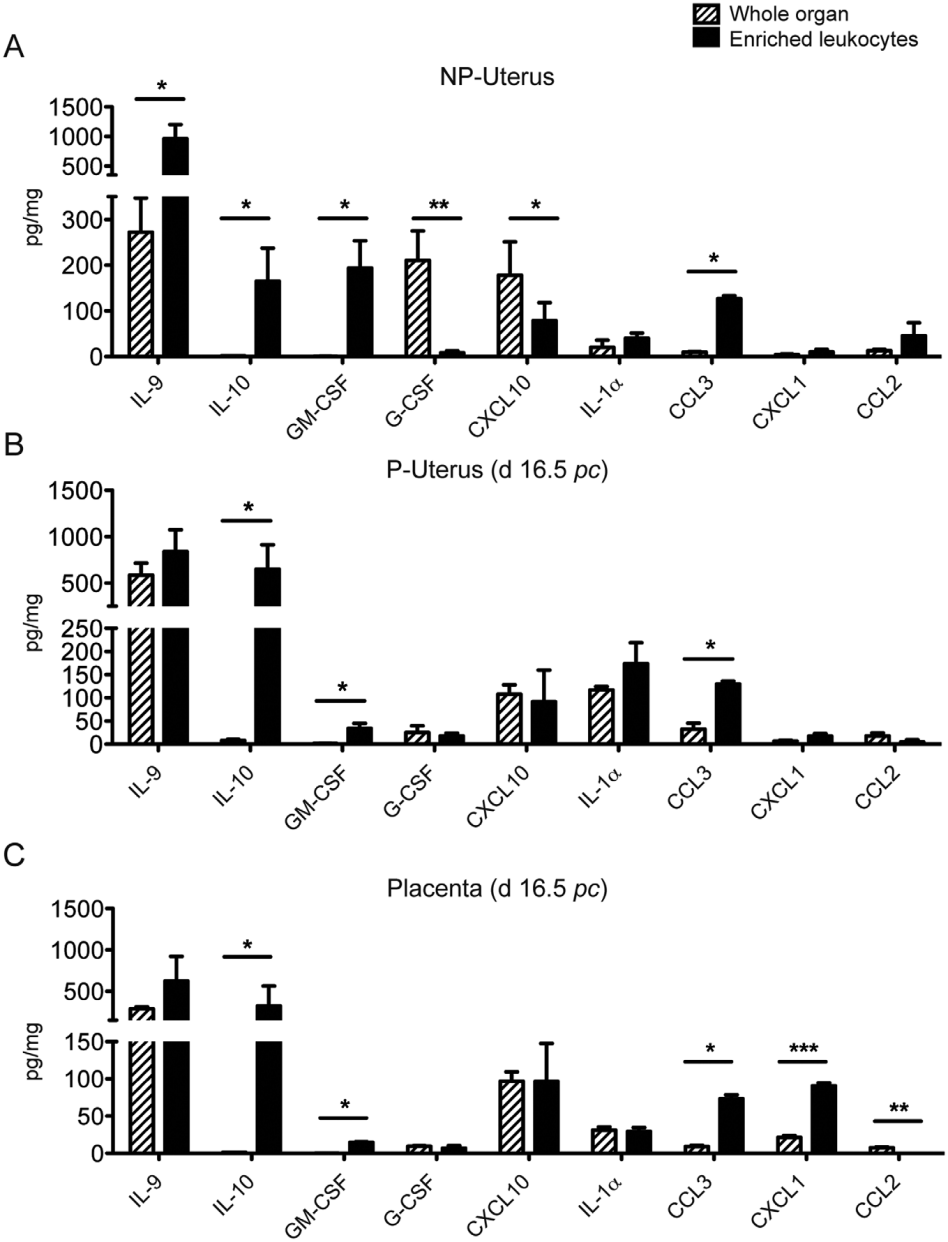
Quantification of uterine and placental cytokines and chemokines in syngeneic pregnancy (day 16.5 *pc*). Uterine and placental tissues were prepared as described in Materials and Methods : (A) Uteri from NP mice, (B) Uteri from **syngeneic** pregnancy (day 16.5 d*pc*), (C) Placenta from **syngeneic** pregnancy (same animals). Samples were analyzed simultaneously for the following 22 cytokines: IL-1α, IL-1β, IL-2, IL-4, IL-5, IL-6, IL-7, IL-9, IL-10, IL-12(p70), IL-13, IL-15, IL-17, CSF2 (GM-CSF), CSF3 (G-CSF), IFNγ, CXCL10 (IP-10), CXCL1 (KC), CCL2 (MCP-1), CCL3 (MIP-1α), CCL5 (RANTES), TNF-α. Only IL-9, IL-10, GM-CSF (CSF2), G-CSF (CSF3), CXCL10 (IP-10), IL-1α, CCL3 (MIP-1α), CXCL1 (KC), CCL2 (MCP-1) yielded reproducibly significant measurements and have been presented. Striped bars: whole organ, black bars: enriched leukocytes from the same organ. Data are from 4 to 5 different samples. Statistically significant differences: (*) p≤0.05, (***) p≤0.001.

It is well known that the local cytokine and chemokine milieu is critical for maintaining a successful pregnancy, although no detailed picture has emerged yet. Therefore, we have quantified 22 cytokines simultaneously (IL-1α, IL-1β, IL-2, IL-4, IL-5, IL-6, IL-7, IL-9, IL-10, IL-12 (p70), IL-13, IL-15, IL-17, CSF2 (GM-CSF), CSF3 (G-CSF), IFN-γ, CXCL10 (IP-10), CXCL1 (KC), CCL2 (MCP-1), CCL3 (MIP-1α), CCL5 (RANTES), TNF-α) in lysates from NP or pregnant uterus or placenta, as well as from enriched uterine leukocytes isolated from the same tissues. The results are summarized in Figure 7, and compiled in Figure 5, on the same criteria of similarities and differences as applied to leukocyte percentages. We have presented cytokine or chemokine values for which we had obtained at least 4 significant measurements over background.

Interestingly, in NP mice, IL-9 was one of the most abundant uterine cytokines measured (note the scale differences) in whole organ (272±75 pg/mg) and the most abundant in enriched uterine leukocytes (964±237 pg/mg), suggesting it is produced in majority by leukocytes (Figure 7A). In whole NP uterus, other cytokines of important quantity were G-CSF (211±64 pg/mg), which is barely detectable in uterine leukocyte (8±4.6 pg/mg) and CXCL10 (178±73 pg/mg) again significantly less abundant in leukocytes than in whole organ, indicating that most of G-CSF and around 50% of CXCL10 are produced in majority by stromal cells (Figure 7A).

The next most abundant cytokines, after IL-9, detected primarily in NP uterine leukocytes were GM-CSF (194±60 pg/mg), IL-10 (165±72 pg/mg), CCL3 (127±6 pg/mg), CXCL10 (79±39 pg/mg) and CCL2 (46±28 pg/mg) (Figure 7A).

Our results indicate that enriched leukocytes from a NP uterus are the main source of IL-9, the most abundant cytokine we have measured. They also produce IL-10, GM-CSF, CCL3 and CCL2 (Figure 7A and Figure 5E,F), whereas uterine stromal or epithelial cells mainly produce G-CSF and CXCL10 (Figure 7A). Other cytokines analysed were barely detectable in whole NP uterus and resident leukocytes (data not shown).

During a syngeneic pregnancy (day 16.5 *pc*), IL-9 was again the most abundant cytokine measured in uterus (585±129 pg/mg) or enriched leukocytes (839±235 pg/mg) (Figure 7B, Figure 5G) (again note the scale differences, Figure 7B). In contrast, IL-10 expressed in the same range as IL-9 (650±263 pg/mg, Figure 5G) in enriched leukocytes was barely detectable in whole organ suggesting strongly that leukocytes were the main source of IL-10 (Figure 7B). In pregnant uterus, the next most expressed cytokines were IL-1α (117±7 pg/mg), CXCL10 (108±21 pg/mg) and G-CSF (25±14 pg/mg) expressed in comparable ranges in enriched leukocytes (173±45, 92±68 and 18±6 pg/mg, respectively). CCL3 (32±13 pg/mg) detectable in pregnant uterus was again significantly more abundant in enriched leukocytes (130±6 pg/mg).

In day 16.5 *pc* placenta (Figure 7C), IL-9 was again found at highest levels both in whole organ (291±24 pg/mg) and placental leukocytes (624±297 pg/mg, Figure 5G) and IL-10 was only detectable in leukocytes, although in smaller amounts than IL-9 (325±237 pg/mg, Figure 5G). CXCL10 (96±51 pg/mg), and IL-1α (29±5 pg/mg) were equally distributed between the whole placenta and placental leukocytes, and in the same dose range as in pregnant uterus (Figure 7C, Figure 5G). G-CSF is also present in placenta (9.6±0.9 pg/mg) and leukocytes (7±3 pg/mg) but in smaller quantities (Figure 7C). In contrast, CXCL1 (92±4 pg/mg), CCL3 (73±7 pg/mg) and GM-CSF (15±1 pg/mg) are clearly enriched in leukocytes, (p≤0.05) (Figure 7C, Figure 5H) and CCL2 is mainly produced by stromal cells.

Our data indicate that, similarly to the uterus, IL-9 is the most abundant cytokine, among the 22 tested, present in the placenta, both in whole organ or enriched leukocytes. IL-1α and CCL3 are present in larger quantities in pregnant uterus than in placenta. IL-9, IL-10 and CXCL10 are present in comparably large quantities both in pregnant uterus and in placenta (Figure 2B, 2C, Figure 5G). In contrast, placental leukocytes produce more CXCL1 than their uterine counterparts (Figure 2B, 2C, Figure 5H).

### Cytokine and chemokine expression levels in uterus from NP versus syngeneic or allogeneic pregnancies

**Figure 8:**
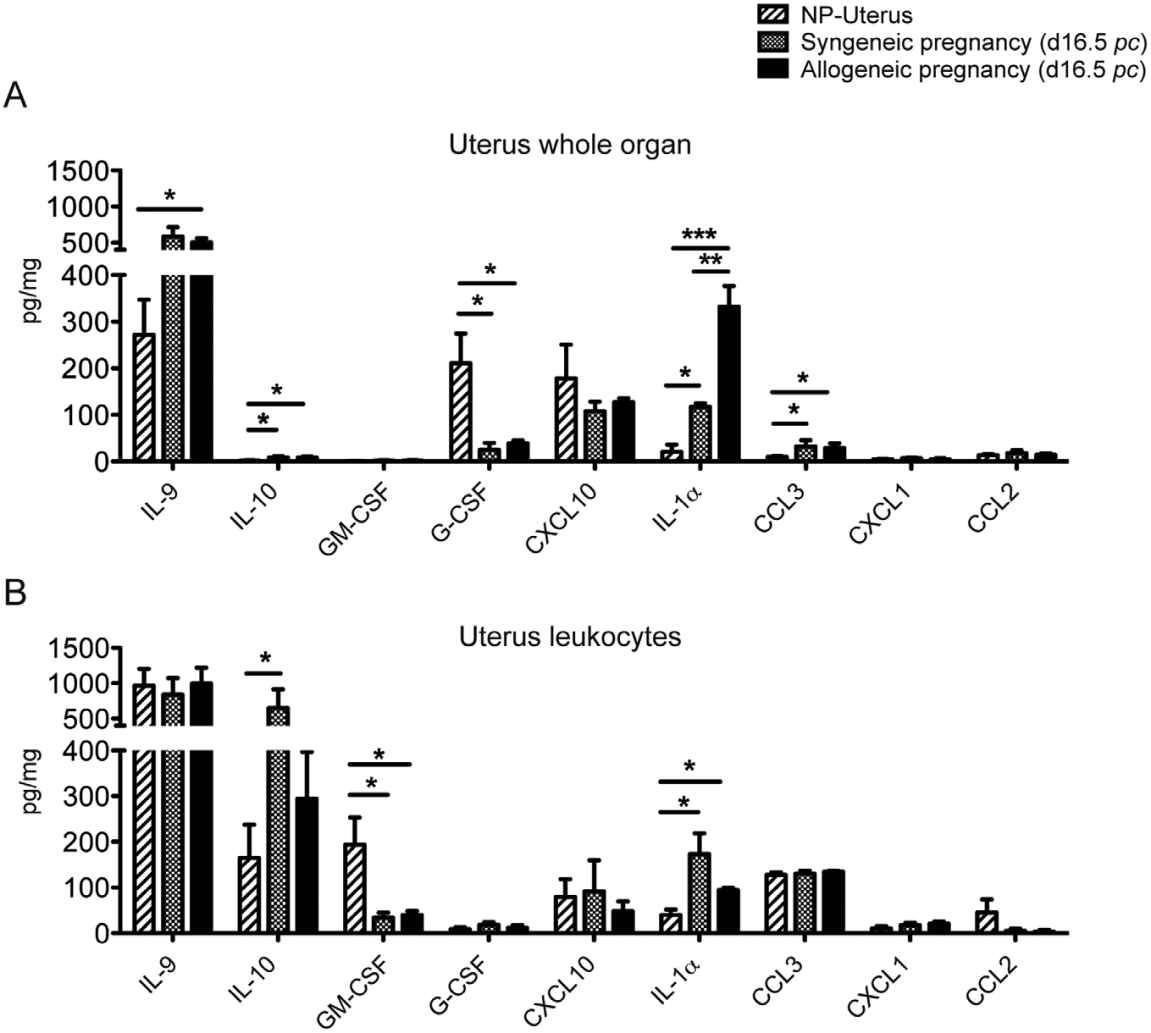
Cytokine and chemokine expression levels in uterus from NP mice versus syngeneic or allogeneic pregnancies (day 16.5 *pc*). The same protocol as in Figure 7 was followed. Proteins were measured from whole uterus (A), or from enriched uterine leukocytes (B) from NP uterus, or syngeneic or allogeneic pregnancies (note the important scale variations). Striped bars: NP uterus, grey bars: syngeneic pregnancy at 16.5 d*pc*, black bars: allogeneic pregnancy at 16.5 d*pc*. Data are from 4 to 5 different samples. (*) p≤0.05, (**) p≤0.005, (***) p≤0.001.

The experiment presented in Figure 7 has been confirmed and extended to compare NP, and syngeneic versus allogeneic pregnancies. The results are presented in Figure 8.

The amount of uterine IL-9 in the whole NP uterus increases during pregnancy (Figure 8A), although a statistically significant difference is seen in allogeneic crosses only (by 86%, from 272±75 pg/mg in NP uterus to 507±56 pg/mg in allogeneic pregnancy, p≤0.05). In contrast, G-CSF decreases from 211±64 pg/mg in a NP uterus, to 25 pg/mg (down by more than 80% in both syngeneic and allogeneic pregnancies) (Figure 8A, Figure 5F). CXCL10, one of the most abundant cytokine in the whole uterus is stable at 178±73 pg/mg in NP uterus, 107±21 pg/mg in syngeneic, and 127±8 pg/mg in allogeneic pregnancy (Figure 8A). Remarkably, IL-1α is augmented by 485% in syngeneic pregnancy and 1560% in allogeneic pregnancy (p≤0.001). CCL3 is also significantly increased both in syngeneic and allogeneic situations, although on a smaller scale (Figure 8A).

Figure 8B presents the cytokine content in enriched uterine leukocytes from NP mice (hatched bars), and pregnant mice from syngeneic (light grey bars), or allogeneic (black bars) crosses. In contrast to the whole uterus, the leucocyte content in IL-9 was similarly high in the three situations (between 900 and 1000 pg/mg, Figure 8B, Figure 5E and G). Compared with the NP situation, IL-10 is significantly augmented (294%) in leukocytes from a syngeneic pregnancy only (p≤0.05, Figure 8B, Figure 5F), and it is also increased by 78% in an allogeneic gestation, and IL-1α significantly increases during pregnancy in both allogeneic and syngeneic crosses (by 340 and 140% respectively, (p≤0.05, Figure 8B, Figure 5F).

CCL3, CXCL10, G-CSF, CXCL1 and CCL2 are stable in the 3 different conditions (Figure 8B and Figure 5E). In contrast, GM-CSF is drastically decreased by about 80% in either cases of pregnancy (p≤0.05, Figure 8B, Figure 5F). These results reveal that pregnancy induces major alterations in the expression pattern of cytokines in the uterus. When we compared NP and pregnant conditions, we found a significant increase in the IL-1α expression level in syngeneic pregnancy and an even more drastic increase during allogeneic pregnancy. IL-9, IL-10 and CCL3 expression levels increase also during pregnancy but less drastically. In contrast, we observed a sharp significant decrease in G-CSF in whole uterus, and of GM-CSF in uterine leukocytes. Finally our data indicate that syngeneic and allogeneic pregnancies only differ significantly in the quantities of IL-1α present in whole uterus.

### Cytokine and chemokine expression levels in placenta from syngeneic or allogeneic pregnancies

Placental contents in cytokines and chemokines (whole organ and enriched leukocytes) were measured from the same mice as in Figure 8.

**Figure 9:**
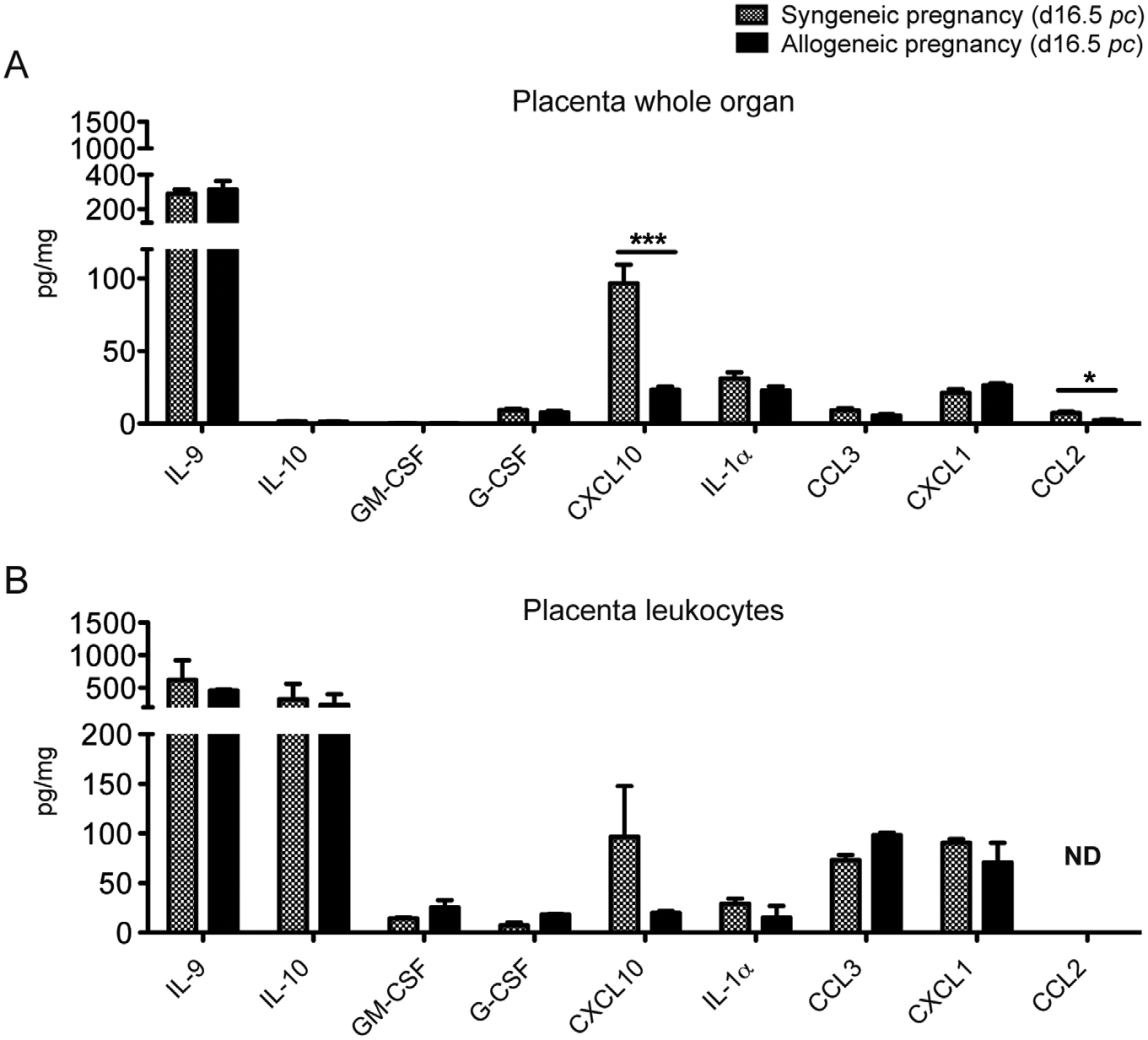
Cytokine and chemokine expression levels in placentae from syngeneic or allogeneic pregnancies (day 16.5 *pc*). Analyses were performed on the placentas from the same pregnant mice as in Figure 8. Proteins were measured from whole placenta (A), or from enriched placental leukocytes (B) from syngeneic or allogeneic pregnancies (note the important scale variations). Grey bars: syngeneic pregnancy, black bars: allogeneic pregnancy. Data are from 4 to 5 different samples. (*) p≤0.05, (**) p≤0.005, (***) p≤0.001.

As shown in Figures 9A and 9B, the majority of placental cytokine measurements were strictly comparable in syngeneic or allogeneic pregnancies, with IL-9 quantities being much higher on the scale than any other cytokine measured. IL-10 is highly expressed but in enriched placental leukocytes only (Figure 9B).

In whole placenta, CXCL10 and CCL2 were significantly less produced, by 75% and 67%, respectively, in an allogeneic gestation compared to a syngeneic one. We did not see any other significant difference for all other cytokines measured, including IL-9. The two major cytokines produced by placental leukocytes, IL-9 and IL-10, are not significantly altered in an allogeneic gestation (Figure 9B). CXCL10 is present at high levels (96±51 pg/mg) in placental leukocytes from a syngeneic pregnancy. Although it is also drastically decreased (79%) in an allogeneic pregnancy, the difference is not statistically significant, due to important variations between individual samples from syngeneic pregnancies (Figure 9B).

## Experimental Procedures

### Mice

C57BL/6 (B6) mice were purchased from Charles River (France) and CBA/Ca mice from Harlan (Belgium). Female and male mice (8–12 week old) were housed in specific pathogen-free conditions.

All animal work has been conducted according to relevant European and NIH institutional guidelines. The use of mice in the manuscript was approved by the local Institutional Animal Care and Use (IACUC) and ethics Committee, officially referred to as “Animal Experimentation Ethical Committee, Buffon” (CEEA-40). The approved protocol number is CEB-05-2012. The current version of this protocol is valid until March 29 2015.

### Pregnancy

Naturally cycling virgin B6 females were caged with either B6 or CBA/Ca males. Mating was assessed by the presence of a vaginal plug. The day of detection of the vaginal plug was defined as 0.5 d*pc*. The day of delivery was defined as day 0.5 d*pp*.

### Isolation of uterine and placental tissues or leukocytes

At 5.5; 10.5; 13.5; 16.5 d*pc* and 0.5; 2.5; 5.5 d*pp*, female mice (and NP ones as controls) were exsanguinated by full cardiac puncture after CO_2_ anesthesia, to minimize maternal blood contamination, which we checked on uterus/placenta paraffine sections stained by Hematoxylin/Eosin (Figure S3). In earlier series of experiments, anesthetized individual mice were perfused with 40 to 50 ml of Phosphate Buffered Saline (PBS), but we did not see significant changes in the resulting cell populations compared to exsanguination.

At 5.5 d*pc* and 0.5; 2.5; 5.5 d*pp*, whole uteri were excised and processed as described below. At 10.5; 13.5; 16.5 d*pc,* uteri and separate placentas were excised and further processed as described below.

### Preparation of placental tissue and leukocytes

We used pregnant mice on days 16.5 d*pc* when the placenta is fully mature and fetal-maternal interactions and exchanges are most active. The placentas were separated from extra-embryonic tissues, umbilical cord and fetus, cut in small pieces and gently crushed on nylon filter (Cell strainer Falcon; BD Biosciences, Ref. 352350) and then rinsed with ice-cold Phosphate Buffer Saline (PBS) (Gibco; Ref. 14190-094). The cell suspension was centrifuged at 280 g for 5 minutes. We then lysed red blood cells with ammonium-chloride lysis buffer for 1 minute and washed the cells with a high volume of Flow cytometry (FC) buffer composed of PBS-Fetal Calf Serum (FCS) 4% (Gibco; Ref. 10270-106)- Sodium azide (N_3_Na) (Sigma-aldrich; Ref S2002-500G) 0.1% (FC buffer). After a 5 minutes centrifugation at 280 g, pelleted cells were resuspended in 80% Percoll (GE Healthcare Bio-Sciences AB, Ref. 17-0891-01). A 40% Percoll solution was then added delicately above the cell suspension and the gradient was centrifuged at 1510 g for 30 minutes (15°C) without break. Leukocyte-enriched cells recovered from the interface between the two Percoll layers were washed in a large volume of FC buffer, spun at 280 g 5 minutes and resuspended in FC buffer to be kept on ice and counted.

### Preparation of uterine tissue and leukocytes

Uteri from NP or *post partum* mice, and from 5.5 d*pc* pregnant mice were excised and all surrounding tissue and fat were removed. At 10.5; 13.5; 16.5 d*pc,* fetal-placental units were gently detached from their implantation sites within the uterine wall. Whole uteri were cut into small pieces and incubated in RPMI 1640 containing Collagenase A (0.875 mg/ml; Roche, Ref. 103 586), 16% FCS and DNase I (10 U/ml; Roche, Ref. 11-284-932-001) for 1 hour at 37°C with intermittent stirring. The supernatant was filtered on a cell strainer 70 mm (BD Falcon; Ref. 352350) and tissues were pushed delicately through and rinsed with FC buffer. Next, we incubated the filtered cell suspension in DNase I (10 U/ml) and 10% FCS for 30 minutes, then centrifuged it at 280 g for 5 minutes. Subsequently, uterine leukocytes were prepared using the same protocol as placental leukocytes. For the measurements of interleukin and chemokine contents, squares around implantation sites were cut out of the uterine wall before processing (see below).

### Flow Cytometry analyses

In order to block non-specific antibody binding and staining, cell aliquots (6.10^4^ per sample) were pre-incubated for 30 minutes with anti-CD16/CD32 monoclonal antibodies, or an immunoglobulin excess from the same species as the antibody used for staining. Cells were washed once with FC buffer and centrifuged at 280 g for 5 minutes. Cell pellets were resuspended and incubated with various combinations of specific antibodies from a panel compiled in Table 1. Predetermined optimal concentrations of antibodies were added to the cell suspensions and incubated for 1 hour on ice. Biotin-coupled antibodies were revealed by fluorochrome-coupled Streptavidin (Table 1). The cells were washed with FC buffer, and resuspended in 250 µl of FC buffer. Propidium Iodide (PI) or Sytox blue (Table 1) was added to each sample to exclude dead cells. Samples were analysed on a CyAn LX flow cytometer (Dako Cytomation) equipped with 405, 488 and 635 nm lasers, or on an LSRII analyzer (Becton Dickinson, France) equipped with 405, 488, 561, and 640 nm lasers. Only viable cell populations were analysed on the basis of forward and side scatter criteria. In every experiment, strict staining specificity controls were performed throughout the study, with species- and isotype-matched immunoglobulins, coupled to the same fluorochromes in a comparable fluorochrome/protein ratio, that were used in equal amounts to the relevant antibodies.

### Cytochemical analysis of NP uterus sorted cells

Viable CD45.2+, CD11b+, F4/80+ R1 (C) or R2 (D) cells were sorted on a FACS Aria III (Becton Dickinson, France) equipped with 405, 488, 561, and 640 nm lasers, and spun onto Super + glass microscope slides using a Thermo Shandon Cytospin 4 cytofuge (4 min at 400 g). Cells were fixed in methanol before staining with Wright-Giemsa. Pictures were taken on a Nikon H600L microscope equipped with a DS-Fi2-Nikon camera.

### Cytokine detection assay

Uterine implantation sites or whole placentas were crushed for 1 minute in ice-cold lysis buffer (Tris-HCl: 20 mM, pH 7.5, MgCl2 5 mM, DTT 1 mM containing protease inhibitor cocktail (Roche; compete, EDTA-free, Ref. 11-873-580-001)), with a Polytron PT 1600E tissue grinder (KINIMATICA AG). The tissue lysates were centrifuged at 14 000 g in a pre-cooled (4°C) centrifuge for 15 minutes. Supernatants were immediately transferred to a new tube and stored at −80°C.

Leukocyte-enriched cells isolated from uterus or placenta were washed twice in ice-cold PBS and pelleted at 280 g for 10 minutes. Ice-cold lysis buffer was added on the cell pellet and sonicated for 1 minute (Branson). The cell lysates were centrifuged at 14 000 g in a pre-cooled centrifuge for 15 minutes. Supernatants were immediately transferred to a new tube and stored at −80°C.

Samples were tested simultaneously for the following 22 cytokines: IL-1α, IL-1β, IL-2, IL-4, IL-5, IL-6, IL-7, IL-9, IL-10, IL-12(p70), IL-13, IL-15, IL-17, CSF2 (GM-CSF), CSF3 (G-CSF), IFN-γ, CXCL10 (IP-10), CXCL1 (KC), CCL2 (MCP-1), CCL3 (MIP-1α), CCL5 (RANTES), TNF-α. This was achieved using a 22-plex fluid-phase immunoassay using custom kits (Millipore; MCYTO-70K-22), run on a LUMINEX 100 IS system (Luminex Corporation, Austin, TX, USA), using xMAP technology detection methods. These assays were performed at the ANEXPLO core facility (IFR 31, Toulouse, France). The lower limit of detection of IL-1α, IL-1β, IL-2, IL-4, IL-5, IL-6, IL-7, IL-12 (p70), IL-13, IL-15, IL-17, GM-CSF (CSF2), G-CSF (CSF3), IFN-γ, CXCL10 (IP-10), CXCL1 (KC), CCL2 (MCP-1), CXCL5 (RANTES), TNF-α was 3.2 pg/ml; 14.7 pg/ml for IL-10; 17.15 pg/ml for CCL3 (MIP-1α) and 80.35 pg/ml for IL-9. The upper detection limit of IL-1α, IL-1β was 5 614.38 pg/ml and 10 000 pg/ml for all other cytokines.

### Statistical analyses

The cell percentage and the cell number per organ followed Gaussian distribution. Thus, all statistical analyses were performed using unpaired Student t tests.

## Discussion

In the present work, we have quantified the different leukocyte populations (innate and adaptive immune cells) in female B6 mice by flow cytometry and analysed a panel of 22 cytokines/chemokines in NP uterus and during syngeneic or allogeneic pregnancy, by Luminex technology. We have used two very sensitive and reproducible techniques. This systematic and detailed analysis brought interesting novel information: 1) in the NP uterus, HGC are mostly composed of eosinophils and they undergo a sharp decline early during pregnancy, with a parallel high local expansion of myeloid cells, NK cell and T cells, granulocytes (neutrophils), B and NKT cells, 2) in striking contrast compared to the uterus, the placenta during a syngeneic gestation, harbours a majority of granulocytes (neutrophils), myeloid Gr-1+ cells including monocytes, and B cells, 3) a comparison of a syngeneic versus an allogeneic gestation reveals no major modifications in the leukocyte distributions in each organ, except in placenta where we measured a significant decline in frequency and numbers of monocytes and myeloid CD11c+ cells, 4) the up to now unreported presence of high quantities of IL-9 in NP, and pregnant uterus and placenta, in larger amounts than the previously described IL-10, 5) the presence of high quantities of CXCL1, and CCL3 and CXCL10 in placental leukocytes, 6) a huge increase in uterine IL-1α and a significant decrease in uterine G-CSF and GM-CSF both in syngeneic and allogeneic pregnancies, 7) a significant decrease in placental pro-inflammatory chemokines CXCL10 and CCL2 in allogeneic compared to syngeneic crosses.

Our results have shown that the leukocyte preparations we performed on day 16.5 *pc* were more than 95% positive for the pan leukocyte marker CD45. Leukocytes of fetal origin were rarely found in the preparations from uterus (<2%) and placenta (<5%). This is not surprising as the first single positive mature CD4+ T cells are generated in the fetal thymus around day 18 *pc* and start emigrating around 3 days after birth. Likewise, B220+CD43- fetal B cells appear in fetal liver around day 18 of gestation. The fetal bone marrow starts producing all hematopoietic cell lineages, including NK cells and monocytes, very shortly before birth [28].

It has been known for a long time that uterine leukocyte populations vary with the phases of estrus cycle [34], [36] but published studies on leukocytes and cytokines/chemokines present at the mouse fetal-maternal interface have not been extensive. In the majority of important studies, authors focused on particular decidual cell types, not on a panel encompassing different leukocyte populations from placenta or uterine implantation sites [2], [29]–[33]. This is probably due to the technical difficulty, compared to lymphoid organs, of preparing viable leukocytes from placenta and uterus with satisfactorily high cell yields. Consequently, the panel of mouse uterine and placental leukocyte populations and cytokines/chemokines, and their fate at the fetal-maternal interface during pregnancy, needed to be better characterized.

In the NP uterus, our results clearly indicated that eosinophilic granulocytes are the main population of HGC in an enriched leukocyte preparation from a pool of NP B6 uteri. We had to pool uteri from 5 to 6 mice to get high enough leukocytes to allow flow cytometry analyses and cell sorting. Eosinophils infiltration in the NP uterus has been observed and confirmed decades ago in different species [35]–[38] but their relative proportion in tissue leukocytes had not been measured so far. It has been postulated that those cells could play a role in preparing the uterus for pregnancy, in blastocyst implantation or in protection against infection [39]. The high amounts of IL-9, IL-10, GM-CSF and CCL3 (MIP-1α) we have measured in this study in enriched leukocytes is possibly the consequence of eosinophil secretion [39], as they have a pro-inflammatory activity and are responsible for tissue homeostasis [40]. Interestingly, G-CSF and GM-CSF are present in NP uterus, and decrease significantly during pregnancy, both in syngeneic and allogeneic crosses. This drop correlates with the disappearance of eosinophilic granulocytes, which suggests that G-CSF and GM-CSF could play an important role to recruit and maintain this leukocyte population before or during implantation [41]. Our results correlate with data from others showing that G-CSF- and GM-CSF-deficient mice have impaired reproductive capacities [6], [42]. In contrast, higher concentrations of G-CSF- and GM-CSF might be harmful for pregnancy because of their pro-inflammatory properties. The lower dose of GM-CSF found in pregnant uterus could help maintaining maternal tolerance and avoiding fetal rejection.

Another finding was the very significant increase of uterine IL-1a during pregnancy, particularly in allogeneic crosses. IL-1α and IL-1β are among the most potent host proteins that activate inflammatory responses to both danger-associated molecular patterns (DAMPs) and pathogen-associated molecular patterns (PAMPs) [43]. This cytokine has been shown to contribute to fetal death following LPS administration in pregnant mice [44].

These important differences in uterine leukocyte population and cytokines/chemokines present during pregnancy compared to NP uterus postulate that hormonal stimulation as well as remodelling due to decidualisation and implantation, were probable causes and/or consequences of these modifications. We have found that myeloid CD11c+ cells, myeloid GR1+ cells including monocytes and NK cells represent the 3 major cell types in a pregnant uterus. They were most probably drawn at the sites of implantation to mature and proliferate locally as shown by J. Pollard’s group for macrophages [45] and A. Croy’s laboratory for major roles played by uterine NK cells [29], [46]–[49].

Another striking observation is the few significant differences observed between syngeneic and allogeneic pregnancies. During evolution, numerous mechanisms have been selected, in parallel to the evolutions of the mammal immune system and environmental pathogens, to maintain the maternal tolerance to the semi-allogeneic fetus. The mechanisms of cellular and cytokine/chemokine interactions, and their regulations, have evolved in a context of allogeneic pregnancy. Our hypothesis is that these mechanisms are conserved and operate even in an experimental syngeneic pregnancy especially in a non-challenging SPF environment.

Interestingly, the placenta contains a majority of granulocytes, myeloid GR1+ cells including monocytes and B cells. These results are in agreement with published results in the mouse where a preferential increase of uterine monocytes over other cell types has been observed in pregnancy [50], [51]. Likewise, Blois *et al.* [52] have found an increase in CD11c^+^ uterine cells (mostly myeloid DC) isolated by tissue digestion during gestation. Our results are also in agreement with the small frequency and numbers of NKT cells found at the fetal-maternal interface by Ito *et al.* [51], [53]. However, in the allogeneic situation, placental myeloid Gr-1+ cells, including monocytes/macrophages, and myeloid CD11c+ cells were significantly reduced. This may diminish antigen-presenting capacity to naïve T cells in the placenta, preventing paternal alloantigen stimulation of maternal T cells, thus protecting the fetus against deleterious maternal immune responses. During allogenic pregnancy, we also observed a decrease in placental CCL2 and CXCL10, two important chemokines involved in the regulation of lymphocyte/monocytes recruitment [54]–[56], and associated with allograft rejection [57]. The role of CXCL10 and CCL2 is emerging as regulators of the recruitment of inflammatory cells into tissues during inflammatory responses [58]. Their inhibition may be beneficial in different clinical scenarios [57], including pathologies due to stress or infection during pregnancy.

It is also interesting to notice that IL-9 and IL-10 are the most abundant placental cytokines detected in our panel followed by CXCL10, CXCL1, CCL3, IL-1α and CCL2 (Figure 5). As expected, we found that IL-10, a Th2 cytokine known to be present at the fetal-maternal interface, was not only present in high quantity in NP uterus but was significantly increased in syngeneic and allogeneic pregnancy, both in the uterus and the placenta. This is in agreement with a previous report on syngeneic pregnancy based on RT-PCR methods [59]. However, White *et al.* [60] have shown that IL-10 is not essential for maternal immune tolerance or a successful pregnancy. In order to uncover the role of IL-10, Murphy *et al*. [61] showed that IL-10 plays an important protective role during pregnancy following a stress challenge such as the injection of low doses of LPS. In the absence of IL-10, fetal resorptions were observed in association with cytotoxic uNK cell activation and TNFα production. In contrast, we found that IL-4 was undetectable at the fetal maternal interface, which differs from RT-PCR data on placental tissue published by Delassus *et al.* [59]. This discrepancy is likely to be due to the different methods used (measurement of mRNA by RT-PCR versus detection of proteins by Luminex) and the kinetic of measurement, as they performed analysis on days 12 and 18 *pc*, in placenta versus peripheral blood only.

The presence of large amounts of IL-9 in uterus and placenta was unexpected, as in other Th2 tissues such as lung or gastrointestinal tract, IL-9-producing T cells appeared to have a pro-inflammatory influence [62]. More recently, using IL-9-fluorescent reported mice, it has been shown that Th9 and type 2 innate lymphoid cells (ILC2s) were major sources of worm infection-induced IL-9 production [63]. Interestingly, Fallon *et al.* [64] have shown that despite a lack of IL-4, IL-5, IL-9 and IL-13 gene activity and compromised Th2 responses, female mice reproduce successfully even during an allogeneic pregnancy [65]. IL-9 is a pleiotropic cytokine with direct and indirect effects on many cell types involved in immunity and inflammation [62], [66], [67]. IL-9 seems to be required for allergic inflammation in the lung as well as other tissues, but it also provides protective immunity to intestinal parasites [63]. It is important to note that IL-9 also has been shown to protect mice from a Gram-negative bacterial shock, and the protective effect was correlated with decreases in the production of Th1 cytokines (TNFα, IL-12 and IFNγ), as well as the induction of IL-10 [68], [66]. It would be interesting to investigate the role of IL-9 during pregnancy in infection or inflammation conditions. IL-9 is a member of the γc chain family of cytokines. Cellular sources are Th9 cells, Th2 cells, Th17 cells, Treg cells, mast cells, NK cells. The IL-9 receptor has been characterized in mice and human and has a high affinity for IL-9. The role(s) of IL-9 at the fetal-maternal interface remain to be elucidated. One can only speculate from known IL-9 targets that include mast cells, Treg cells, Th17 cells and antigen-presenting cells [62]. IL-9 might promote Treg cell suppressive functions, and TGFβ production by APC, and thus prevent maternal immune activation against the fetus. We hypothesize that the presence of IL-9 in NP uterus and during pregnancy could also provide protective immunity to infection and/or could down-regulate inflammatory cytokines and up-regulate anti-inflammatory cytokines in conditions of stress or infection, in order to avoid abortion or fetal damage.

In conclusion, our data give the first quantitative and qualitative detailed overview of the different leukocyte populations and cytokines present in NP or pregnant uterus and placenta in B6 mice. Studying the local cytokine milieu, we discovered the presence of surprisingly large amounts of IL-9, and thus revealed a possibly important new player in the regulations at work during pregnancy: down-regulating pro-inflammatory mediators, as well as up-regulating anti-inflammatory cytokines such as IL-10. Th2 and Th9 cytokines could be particularly important in conditions of stress or infectious challenge during pregnancy.

## Supporting Information

Figure S1 **Further characterization of R2-gated, live Gr1-,CD45-2+,CD11b+, CD11c+ cells from syngeneic pregnancy (day 16.5** ***pc*** **).** The same experiment was performed as in Figure 3 of the manuscript. R2-gated, live Gr1-,CD45-2+,CD11b+,CD11c+ cells from placenta (upper line) or pregnant uterus (lower line) were double-stained with fluorochrome-coupled antibodies anti-CD11b along with F4/80, CCR2 or MHC class II markers (Table 1). The experiment was performed twice on pools of 5 to 6 mice.
(TIFF)

Figure S2 **Dolichos Biflorus Agglutinin (DBA) staining of non-pregnant or pregnant uterine R1- and R2-gated leukocytes.** Enriched leukocyte preparations from B6 or B6.RAG2-KO non-pregnant uterus or from B6.RAG2-KO pregnant (10.5 d*pc*) uterus were analyzed by flow cytometry. Viable cells excluding propidium iodide were gated on the basis of forward (FSC) and side scatter (SSC) criteria. Gates R1 (upper line of quadrants) and R2 (lower line of quadrants) were identical to those used in Figures 2, 3 and 4 of the manuscript. Double staining was performed using DBA coupled to biotin and streptavidin-APC and PE-coupled anti-NK1.1 monoclonal antibody (Cf. Materials and Methods). The experiment was reproduced twice on pools of 2 to 3 mice.
(TIFF)

Figure S3 **Histological image of day 16.5** ***pc*** **implant site.** B6 female mice were mated with B6 males. Pregnant females (day 16.5 *pc*) were anesthesized by CO_2_ and exsanguinated by intra-cardiac puncture (average blood volume recovered: 1.5 ml). Freshly collected implant sites were fixed in 4% (wt/vol) paraformaldehyde at 4°C and embedded in paraffin. Seven µm sections were stained with Haematoxylin/Eosin and observed under a LEICA DM2000 light microscope. Lab., labyrinth zone; Spgt, spongiotrophoblast; Mat. Dec., maternal decidua; Myo., myometrium. Arrow: maternal blood lacuna; Blue arrowhead: fetal blood vessel; Black arrowhead: trophoblast giant cell. Scale bar: 200 µm.
(TIFF)

## References

[1] Medawar PB (1953) Some immunological and endocrinological problems raised by the evolution of viviparity in vertebrates. Symp Soc Exp Biol 7: 320–338.

[2] Erlebacher A (2013) Immunology of the maternal-fetal interface. Annu Rev Immunol 31: 387–411. 23298207 10.1146/annurev-immunol-032712-100003

[3] Lavialle C, Cornelis G, Dupressoir A, Esnault C, Heidmann O, et al (2013) Paleovirology of ‘syncytins’, retroviral env genes exapted for a role in placentation. Philos Trans R Soc Lond B Biol Sci 368: 20120507. 23938756 10.1098/rstb.2012.0507 PMC3758191

[4] Dimitriadis E, White CA, Jones RL, Salamonsen LA (2005) Cytokines, chemokines and growth factors in endometrium related to implantation. Hum Reprod Update 11: 613–630. 16006437 10.1093/humupd/dmi023

[5] Moffett-King A (2002) Natural killer cells and pregnancy. Nat Rev Immunol 2: 656–663. 12209134 10.1038/nri886

[6] Ingman WV, Jones RL (2008) Cytokine knockouts in reproduction: the use of gene ablation to dissect roles of cytokines in reproductive biology. Hum Reprod Update 14: 179–192. 18063609 10.1093/humupd/dmm042

[7] Croy BA, Chen Z, Hofmann AP, Lord EM, Sedlacek AL, et al (2012) Imaging of vascular development in early mouse decidua and its association with leukocytes and trophoblasts. Biol Reprod 87: 125. 22954796 10.1095/biolreprod.112.102830 PMC3509781

[8] Yadi H, Burke S, Madeja Z, Hemberger M, Moffett A, et al (2008) Unique receptor repertoire in mouse uterine NK cells. J Immunol 181: 6140–6147. 18941204 10.4049/jimmunol.181.9.6140

[9] Chen Z, Zhang J, Hatta K, Lima PD, Yadi H, et al (2012) DBA-lectin reactivity defines mouse uterine natural killer cell subsets with biased gene expression. Biol Reprod 87: 81. 22875907 10.1095/biolreprod.112.102293 PMC3467293

[10] Lima PD, Croy BA, Degaki KY, Tayade C, Yamada AT (2012) Heterogeneity in composition of mouse uterine natural killer cell granules. J Leukoc Biol 92: 195–204. 22566570 10.1189/jlb.0312136

[11] Bulmer JN, Lash GE (2005) Human uterine natural killer cells: a reappraisal. Mol Immunol 42: 511–521. 15607807 10.1016/j.molimm.2004.07.035

[12] Plaks V, Birnberg T, Berkutzki T, Sela S, BenYashar A, et al (2008) Uterine DCs are crucial for decidua formation during embryo implantation in mice. J Clin Invest 118: 3954–3965. 19033665 10.1172/JCI36682 PMC2582932

[13] Moldenhauer LM, Keenihan SN, Hayball JD, Robertson SA (2010) GM-CSF is an essential regulator of T cell activation competence in uterine dendritic cells during early pregnancy in mice. J Immunol 185: 7085–7096. 20974989 10.4049/jimmunol.1001374

[14] Erlebacher A (2013) Mechanisms of T cell tolerance towards the allogeneic fetus. Nat Rev Immunol 13: 23–33. 23237963 10.1038/nri3361

[15] Nancy P, Tagliani E, Tay CS, Asp P, Levy DE, et al (2012) Chemokine gene silencing in decidual stromal cells limits T cell access to the maternal-fetal interface. Science 336: 1317–1321. 22679098 10.1126/science.1220030 PMC3727649

[16] Wegmann TG, Lin H, Guilbert L, Mosmann TR (1993) Bidirectional cytokine interactions in the maternal-fetal relationship: is successful pregnancy a TH2 phenomenon? Immunol Today 14: 353–356. 8363725 10.1016/0167-5699(93)90235-D

[17] Mitchell MD, Trautman MS, Dudley DJ (1993) Cytokine networking in the placenta. Placenta 14: 249–275. 8367410 10.1016/s0143-4004(05)80426-6

[18] Piccinni MP (2002) T-cell cytokines in pregnancy. Am J Reprod Immunol 47: 289–294. 12148544 10.1034/j.1600-0897.2002.01104.x

[19] Bennett WA, Lagoo-Deenadayalan S, Whitworth NS, Brackin MN, Hale E, et al (1997) Expression and production of interleukin-10 by human trophoblast: relationship to pregnancy immunotolerance. Early Pregnancy 3: 190–198. 10086069

[20] Trautman MS, Collmer D, Edwin SS, White W, Mitchell MD, et al (1997) Expression of interleukin-10 in human gestational tissues. J Soc Gynecol Investig 4: 247–253. 9360229

[21] Hanna N, Hanna I, Hleb M, Wagner E, Dougherty J, et al (2000) Gestational age-dependent expression of IL-10 and its receptor in human placental tissues and isolated cytotrophoblasts. J Immunol 164: 5721–5728. 10820249 10.4049/jimmunol.164.11.5721

[22] Piccinni MP, Beloni L, Livi C, Maggi E, Scarselli G, et al (1998) Defective production of both leukemia inhibitory factor and type 2 T-helper cytokines by decidual T cells in unexplained recurrent abortions. Nat Med 4: 1020–1024. 9734394 10.1038/2006

[23] Lin H, Mosmann TR, Guilbert L, Tuntipopipat S, Wegmann TG (1993) Synthesis of T helper 2-type cytokines at the maternal-fetal interface. J Immunol 151: 4562–4573. 8409418

[24] Jenkins C, Roberts J, Wilson R, MacLean MA, Shilito J, et al (2000) Evidence of a T(H) 1 type response associated with recurrent miscarriage. Fertil Steril 73: 1206–1208. 10856484 10.1016/s0015-0282(00)00517-3

[25] Collins MK, Tay CS, Erlebacher A (2009) Dendritic cell entrapment within the pregnant uterus inhibits immune surveillance of the maternal/fetal interface in mice. J Clin Invest 119: 2062–2073. 19546507 10.1172/JCI38714 PMC2701881

[26] Boring L, Gosling J, Chensue SW, Kunkel SL, Farese RV J, et al (1997) Impaired monocyte migration and reduced type 1 (Th1) cytokine responses in C-C chemokine receptor 2 knockout mice. J Clin Invest 100: 2552–2561. 9366570 10.1172/JCI119798 PMC508456

[27] Serbina NV, Pamer EG (2006) Monocyte emigration from bone marrow during bacterial infection requires signals mediated by chemokine receptor CCR2. Nat Immunol 7: 311–317. 16462739 10.1038/ni1309

[28] Paul W, editor (2008) Fundamental immunology/editor, William E. Paul. 6th ed. Philadelphia: Wolters Kluwer/Lippincott Williams & Wilkins, c2008. 1603 p.

[29] Hofmann AP, Gerber SA, Croy BA (2014) Uterine natural killer cells pace early development of mouse decidua basalis. Mol Hum Reprod 20: 66–76. 24000237 10.1093/molehr/gat060

[30] Kammerer U, Kruse A, Barrientos G, Arck PC, Blois SM (2008) Role of dendritic cells in the regulation of maternal immune responses to the fetus during mammalian gestation. Immunol Invest 37: 499–533. 18716936 10.1080/08820130802191334

[31] Lombardelli L, Aguerre-Girr M, Logiodice F, Kullolli O, Casart Y, et al (2013) HLA-G5 induces IL-4 secretion critical for successful pregnancy through differential expression of ILT2 receptor on decidual CD4(+) T cells and macrophages. J Immunol 191: 3651–3662. 23997222 10.4049/jimmunol.1300567

[32] Barrientos G, Tirado-Gonzalez I, Freitag N, Kobelt P, Moschansky P, et al (2013) CXCR4(+) dendritic cells promote angiogenesis during embryo implantation in mice. Angiogenesis 16: 417–427. 23224220 10.1007/s10456-012-9325-6

[33] Rajagopalan S, Long EO (2012) Cellular senescence induced by CD158d reprograms natural killer cells to promote vascular remodeling. Proc Natl Acad Sci U S A 109: 20596–20601. 23184984 10.1073/pnas.1208248109 PMC3528503

[34] Keenihan SN, Robertson SA (2004) Diversity in phenotype and steroid hormone dependence in dendritic cells and macrophages in the mouse uterus. Biol Reprod 70: 1562–1572. 14766730 10.1095/biolreprod.103.024794

[35] Gansler H (1956) [Electronmicroscopic examination of rat myometrium under the influence of follicular hormones]. Virchows Arch 329: 235–244. 13392508 10.1007/BF00955143

[36] Ross R, Klebanoff SJ (1966) The eosinophilic leukocyte. Fine structure studies of changes in the uterus during the estrous cycle. J Exp Med 124: 653–660. 5950887 10.1084/jem.124.4.653 PMC2138249

[37] Salamonsen LA, Woolley DE (1999) Menstruation: induction by matrix metalloproteinases and inflammatory cells. J Reprod Immunol 44: 1–27. 10530758 10.1016/s0165-0378(99)00002-9

[38] Pepper H, Lindsay S (1960) Levels of eosinophils, platelets, leukocytes and 17-hydroxycorticosteroids during normal menstrual cycle. Proc Soc Exp Biol Med 104: 145–147. 14431670 10.3181/00379727-104-25759

[39] Rothenberg ME, Hogan SP (2006) The eosinophil. Annu Rev Immunol 24: 147–174. 16551246 10.1146/annurev.immunol.24.021605.090720

[40] Chu VT, Beller A, Rausch S, Strandmark J, Zanker M, et al (2014) Eosinophils promote generation and maintenance of immunoglobulin-A-expressing plasma cells and contribute to gut immune homeostasis. Immunity 40: 582–593. 24745334 10.1016/j.immuni.2014.02.014

[41] Robertson SA (2007) GM-CSF regulation of embryo development and pregnancy. Cytokine Growth Factor Rev 18: 287–298. 17512774 10.1016/j.cytogfr.2007.04.008

[42] Seymour JF, Lieschke GJ, Grail D, Quilici C, Hodgson G, et al (1997) Mice lacking both granulocyte colony-stimulating factor (CSF) and granulocyte-macrophage CSF have impaired reproductive capacity, perturbed neonatal granulopoiesis, lung disease, amyloidosis, and reduced long-term survival. Blood 90: 3037–3049. 9376584

[43] Dinarello CA (2009) Immunological and inflammatory functions of the interleukin-1 family. Annu Rev Immunol 27: 519–550. 19302047 10.1146/annurev.immunol.021908.132612

[44] Silver RM, Edwin SS, Umar F, Dudley DJ, Branch DW, et al (1997) Bacterial lipopolysaccharide-mediated murine fetal death: the role of interleukin-1. Am J Obstet Gynecol 176: 544–549. 9077604 10.1016/s0002-9378(97)70545-3

[45] Pollard JW, Lin EY, Zhu L (1998) Complexity in uterine macrophage responses to cytokines in mice. Biol Reprod 58: 1469–1475. 9623608 10.1095/biolreprod58.6.1469

[46] Guimond MJ, Wang B, Croy BA (1998) Engraftment of bone marrow from severe combined immunodeficient (SCID) mice reverses the reproductive deficits in natural killer cell-deficient tg epsilon 26 mice. J Exp Med 187: 217–223. 9432979 10.1084/jem.187.2.217 PMC2212103

[47] Ashkar AA, Di Santo JP, Croy BA (2000) Interferon gamma contributes to initiation of uterine vascular modification, decidual integrity, and uterine natural killer cell maturation during normal murine pregnancy. J Exp Med 192: 259–270. 10899912 10.1084/jem.192.2.259 PMC2193246

[48] Croy BA, Di Santo JP, Greenwood JD, Chantakru S, Ashkar AA (2000) Transplantation into genetically alymphoid mice as an approach to dissect the roles of uterine natural killer cells during pregnancy–a review. Placenta 21 Suppl A: S77–80. 10.1053/plac.1999.0518 10831128

[49] Croy BA, van den Heuvel MJ, Borzychowski AM, Tayade C (2006) Uterine natural killer cells: a specialized differentiation regulated by ovarian hormones. Immunol Rev 214: 161–185. 17100884 10.1111/j.1600-065X.2006.00447.x

[50] Hunt JS, Robertson SA (1996) Uterine macrophages and environmental programming for pregnancy success. J Reprod Immunol 32: 1–25. 8953517 10.1016/s0165-0378(96)88352-5

[51] Habbeddine M, Verbeke P, Delarbre C, Moutier R, Prieto S, et al (2013) CD1d-restricted NKT cells modulate placental and uterine leukocyte populations during chlamydial infection in mice. Microbes Infect 15: 928–938. 23999314 10.1016/j.micinf.2013.08.006 PMC4106417

[52] Blois SM, Alba Soto CD, Tometten M, Klapp BF, Margni RA, et al (2004) Lineage, maturity, and phenotype of uterine murine dendritic cells throughout gestation indicate a protective role in maintaining pregnancy. Biol Reprod 70: 1018–1023. 14681197 10.1095/biolreprod.103.022640

[53] Ito K, Karasawa M, Kawano T, Akasaka T, Koseki H, et al (2000) Involvement of decidual Valpha14 NKT cells in abortion. Proc Natl Acad Sci U S A 97: 740–744. 10639149 10.1073/pnas.97.2.740 PMC15400

[54] Baggiolini M, Dewald B, Moser B (1997) Human chemokines: an update. Annu Rev Immunol 15: 675–705. 9143704 10.1146/annurev.immunol.15.1.675

[55] Schall TJ, Bacon K, Toy KJ, Goeddel DV (1990) Selective attraction of monocytes and T lymphocytes of the memory phenotype by cytokine RANTES. Nature 347: 669–671. 1699135 10.1038/347669a0

[56] Rathanaswami P, Hachicha M, Sadick M, Schall TJ, McColl SR (1993) Expression of the cytokine RANTES in human rheumatoid synovial fibroblasts. Differential regulation of RANTES and interleukin-8 genes by inflammatory cytokines. J Biol Chem 268: 5834–5839. 7680648

[57] Hancock WW, Gao W, Faia KL, Csizmadia V (2000) Chemokines and their receptors in allograft rejection. Curr Opin Immunol 12: 511–516. 11007352 10.1016/s0952-7915(00)00130-8

[58] Hanna J, Goldman-Wohl D, Hamani Y, Avraham I, Greenfield C, et al (2006) Decidual NK cells regulate key developmental processes at the human fetal-maternal interface. Nat Med 12: 1065–1074. 16892062 10.1038/nm1452

[59] Delassus S, Coutinho GC, Saucier C, Darche S, Kourilsky P (1994) Differential cytokine expression in maternal blood and placenta during murine gestation. J Immunol 152: 2411–2420. 8133052

[60] White CA, Johansson M, Roberts CT, Ramsay AJ, Robertson SA (2004) Effect of interleukin-10 null mutation on maternal immune response and reproductive outcome in mice. Biol Reprod 70: 123–131. 13679317 10.1095/biolreprod.103.018754

[61] Murphy SP, Fast LD, Hanna NN, Sharma S (2005) Uterine NK cells mediate inflammation-induced fetal demise in IL-10-null mice. J Immunol 175: 4084–4090. 16148158 10.4049/jimmunol.175.6.4084

[62] Noelle RJ, Nowak EC (2010) Cellular sources and immune functions of interleukin-9. Nat Rev Immunol 10: 683–687. 20847745 10.1038/nri2848 PMC3828627

[63] Licona-Limon P, Henao-Mejia J, Temann AU, Gagliani N, Licona-Limon I, et al (2013) Th9 Cells Drive Host Immunity against Gastrointestinal Worm Infection. Immunity 39: 744–757. 24138883 10.1016/j.immuni.2013.07.020 PMC3881610

[64] Fallon PG, Jolin HE, Smith P, Emson CL, Townsend MJ, et al (2002) IL-4 induces characteristic Th2 responses even in the combined absence of IL-5, IL-9, and IL-13. Immunity 17: 7–17. 12150887 10.1016/s1074-7613(02)00332-1

[65] Miyazaki S, Tanebe K, Sakai M, Michimata T, Tsuda H, et al (2002) Interleukin 2 receptor gamma chain (gamma(c)) knockout mice show less regularity in estrous cycle but achieve normal pregnancy without fetal compromise. Am J Reprod Immunol 47: 222–230. 12069389 10.1034/j.1600-0897.2002.01074.x

[66] Goswami R, Kaplan MH (2011) A brief history of IL-9. J Immunol 186: 3283–3288. 21368237 10.4049/jimmunol.1003049 PMC3074408

[67] Kaplan MH (2013) Th9 cells: differentiation and disease. Immunol Rev 252: 104–115. 23405898 10.1111/imr.12028 PMC3982928

[68] Grohmann U, Van Snick J, Campanile F, Silla S, Giampietri A, et al (2000) IL-9 protects mice from Gram-negative bacterial shock: suppression of TNF-alpha, IL-12, and IFN-gamma, and induction of IL-10. J Immunol 164: 4197–4203. 10754315 10.4049/jimmunol.164.8.4197

