# Supplementary figures and images for: Leukocyte Population Dynamics and Detection of IL-9 as a Major Cytokine at the Mouse Fetal-Maternal Interface
Source: PLoS One. 2014 Sep 26;9(9):e107267. doi: 10.1371/journal.pone.0107267 (PMC4178026; doi:10.1371/journal.pone.0107267)

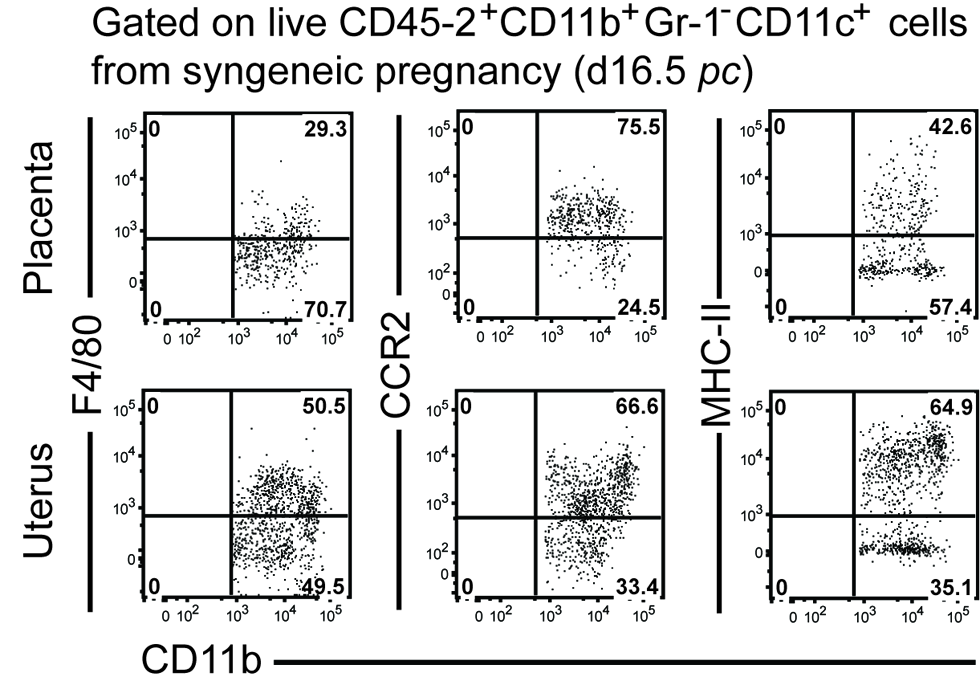

Supplement: Figure S1 — Further characterization of R2-gated, live Gr1-,CD45-2+,CD11b+, CD11c+ cells from syngeneic pregnancy (day 16.5 pc ). The same experiment was performed as in Figure 3 of the manuscript. R2-gated, live Gr1-,CD45-2+,CD11b+,CD11c+ cells from placenta (upper line) or pregnant uterus (lower line) were double-stained with fluorochrome-coupled antibodies anti-CD11b along with F4/80, CCR2 or MHC class II markers (Table 1). The experiment was performed twice on pools of 5 to 6 mice. (TIFF) [file pone.0107267.s001.tiff]

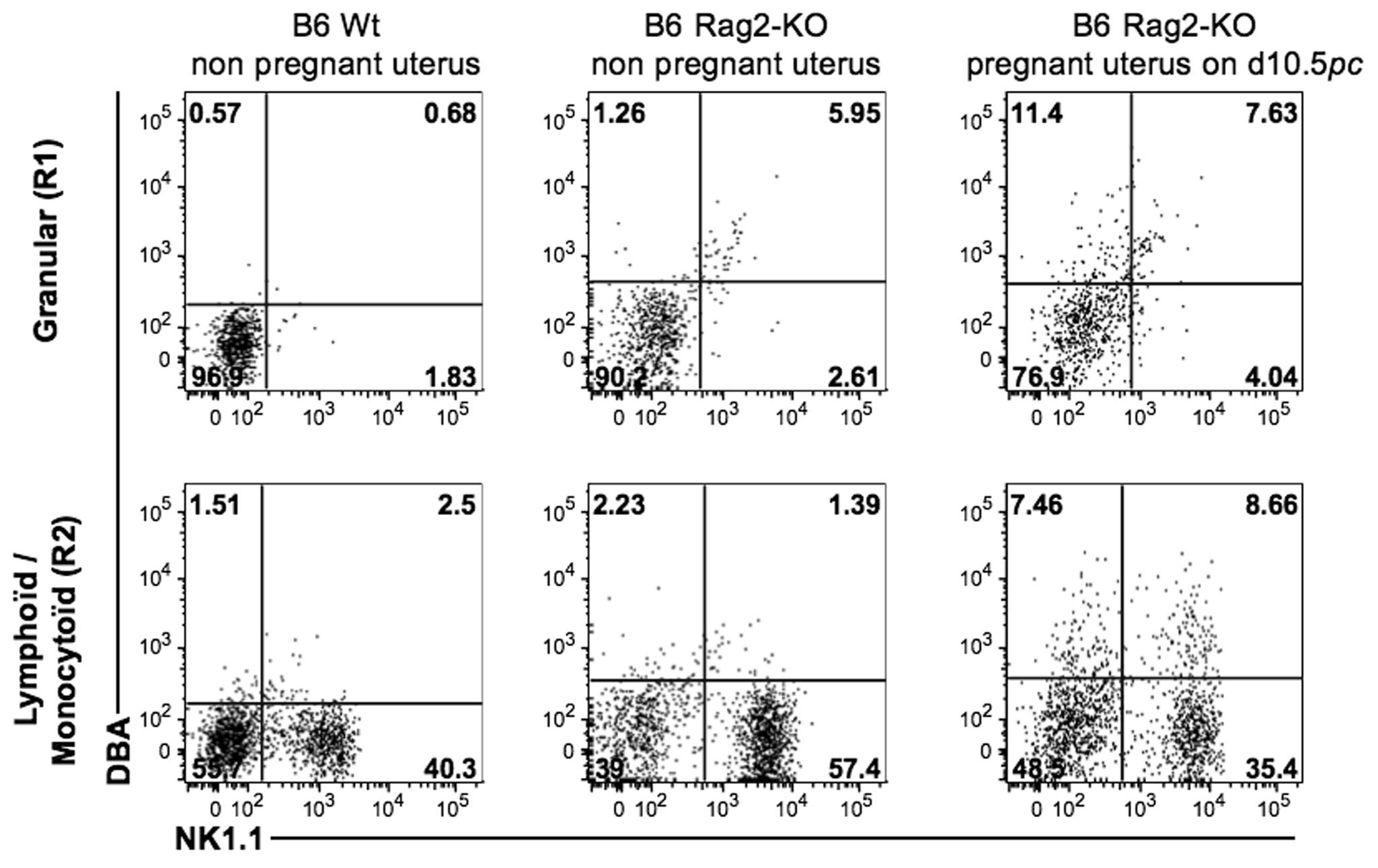

Supplement: Figure S2 — Dolichos Biflorus Agglutinin (DBA) staining of non-pregnant or pregnant uterine R1- and R2-gated leukocytes. Enriched leukocyte preparations from B6 or B6.RAG2-KO non-pregnant uterus or from B6.RAG2-KO pregnant (10.5 dpc) uterus were analyzed by flow cytometry. Viable cells excluding propidium iodide were gated on the basis of forward (FSC) and side scatter (SSC) criteria. Gates R1 (upper line of quadrants) and R2 (lower line of quadrants) were identical to those used in Figures 2, 3 and 4 of the manuscript. Double staining was performed using DBA coupled to biotin and streptavidin-APC and PE-coupled anti-NK1.1 monoclonal antibody (Cf. Materials and Methods). The experiment was reproduced twice on pools of 2 to 3 mice. (TIFF) [file pone.0107267.s002.tiff]

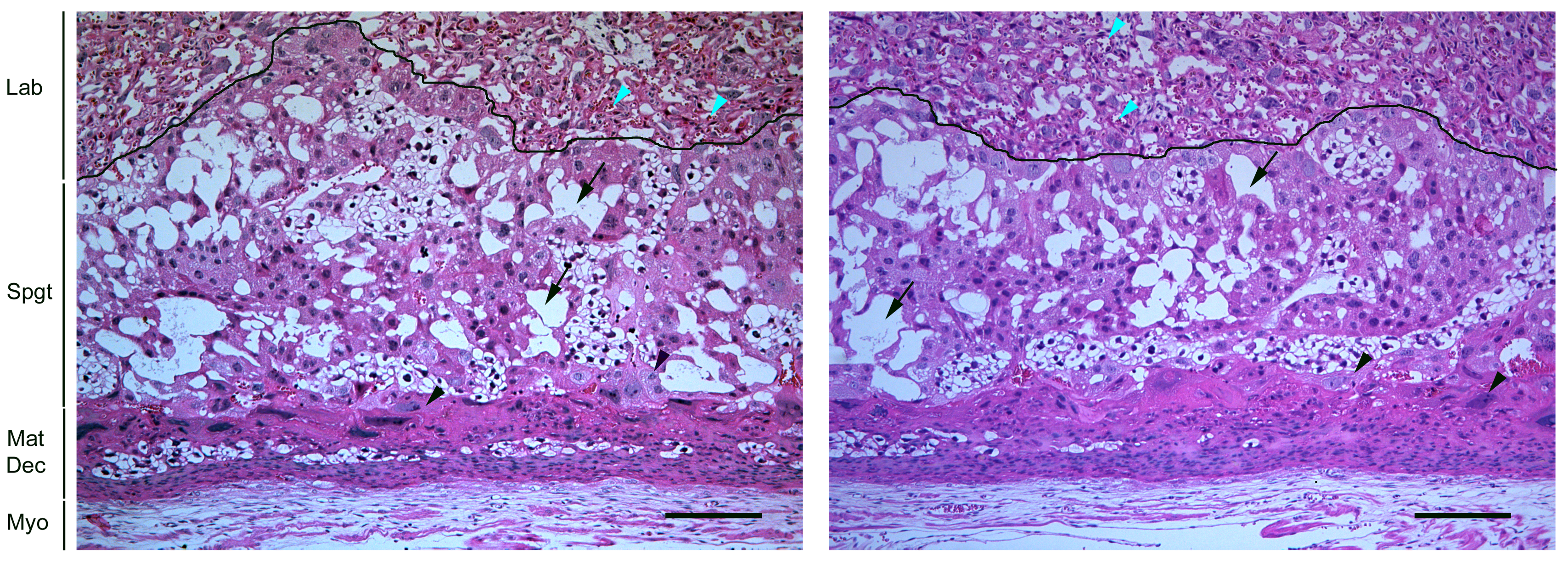

Supplement: Figure S3 — Histological image of day 16.5 pc implant site. B6 female mice were mated with B6 males. Pregnant females (day 16.5 pc) were anesthesized by CO2 and exsanguinated by intra-cardiac puncture (average blood volume recovered: 1.5 ml). Freshly collected implant sites were fixed in 4% (wt/vol) paraformaldehyde at 4°C and embedded in paraffin. Seven µm sections were stained with Haematoxylin/Eosin and observed under a LEICA DM2000 light microscope. Lab., labyrinth zone; Spgt, spongiotrophoblast; Mat. Dec., maternal decidua; Myo., myometrium. Arrow: maternal blood lacuna; Blue arrowhead: fetal blood vessel; Black arrowhead: trophoblast giant cell. Scale bar: 200 µm. (TIFF) [file pone.0107267.s003.tiff]
